# Primitive asteroids as a major source of terrestrial volatiles

**DOI:** 10.1126/sciadv.ado4121

**Published:** 2024-10-11

**Authors:** Rayssa Martins, Elin M. Morton, Sven Kuthning, Saskia Goes, Helen M. Williams, Mark Rehkämper

**Affiliations:** ^1^ Department of Earth Science & Engineering, Imperial College London, London, UK.; ^2^ Department of Earth Sciences, University of Cambridge, Cambridge, UK.

## Abstract

The origins of Earth’s volatiles are debated. Recent studies showed that meteorites display unique mass-independent isotopic signatures of the volatile element Zn, suggesting that Earth’s Zn originated from materials derived from different regions of the Solar System. However, these studies largely omitted meteorites from the differentiated planetesimals thought to represent the Earth’s building blocks, which underwent melting and substantial volatile loss. Here, we characterize the mass-independent Zn isotope compositions of meteorites from such planetesimals. We incorporate these results in mixing models that aim to reproduce Earth’s abundance and isotope compositions of Zn and other elements. Our results suggest that, while differentiated planetesimals supplied ~70% of Earth’s mass, they provided only ~10% of its Zn. The remaining Zn was supplied by primitive materials that did not experience melting and associated volatile loss. Combined with other findings, our results imply that an unmelted primitive material is likely required to establish the volatile budgets of the terrestrial planets.

## Teaser

Primitive unmelted material supplied nearly 90% of Earth’s zinc and likely other volatiles.

## INTRODUCTION

Despite an expanding search in the Solar System and other parts of our galaxy, life remains undetected outside of our planet. With the advancement of observational technology and a seemingly infinite pool of potentially habitable exoplanets, determining the events that led to the habitable conditions on Earth becomes increasingly more important. Yet, both the nature of Earth’s accreting materials and the manner in which they coalesced are still debated.

A common approach to address the origin of the materials from which our planet formed is to use nucleosynthetic isotope anomalies to fingerprint the Earth’s building blocks among diverse Solar System materials. These small but detectable differences (mass-independent “anomalies”) in isotope compositions result from the heterogeneous distribution of isotopically distinct grains through the cloud of gas and dust from which all Solar System bodies formed. An important advantage of this approach is that these anomalies are not altered by secondary stable isotope fractionation induced by physical or chemical processes. This makes them a reliable signature, which can be used to establish genetic links between different materials.

Most meteorite groups in our inventory have been extensively characterized for isotope anomalies of many elements, revealing a clear dichotomy in the distribution of material in the Solar System. Carbonaceous meteorites, which are thought to originate from beyond the orbit of Jupiter, are generally enriched in the neutron-rich isotopes of Fe-peak elements such as Ca, Ti, Cr, Ni, and Zn (*1*–*7*) and depleted in the isotopes produced by slow neutron capture (s-process) of heavier elements such as Sr, Zr, Mo, and Ru (*8*–*11*). In contrast, the inner Solar System, which is sampled by noncarbonaceous (NC) meteorites and terrestrial planets, is characterized by a depletion in the same neutron-rich isotopes and an enrichment in s-process isotopes. Variations are also found within each reservoir to a lesser extent.

The diversity among Solar System materials has been exploited in multiple studies that attempted to reproduce the Earth’s nucleosynthetic isotope composition by modeling mixtures between different meteorite groups. Some of these studies have shown that it is possible to reproduce the Earth’s nucleosynthetic isotope composition for various elements by mixing only chondritic meteorites, which are derived from primitive, undifferentiated planetesimals (*7*, *12*, *13*). Unlike the first generation of planetesimals, these asteroids escaped melting and differentiation because they coalesced after ^26^Al—a short-lived radionuclide whose decay generated substantial heat in the early Solar System—had largely become extinct. Chondrites thus better preserve certain original chemical and physical characteristics, such as high volatile contents, which have largely been lost in early formed differentiated planets and planetesimals. The aforementioned studies therefore largely attempted to reproduce the Earth’s nucleosynthetic isotope composition by mixing carbonaceous chondrites (CCs) with ordinary and enstatite chondrites (OCs and ECs, respectively), which are the two most common types of NC chondrites (NCCs). ECs are also often inferred to account for most of the Earth’s mass because their nucleosynthetic isotope composition is mostly indistinguishable from Earth’s (*1*–*4*, *8*–*11*).

A scenario where Earth accreted primarily from chondrites is, however, not without caveats. The mass-dependent Si and Mg isotope compositions of chondrites are markedly different from the bulk silicate Earth (BSE) (*14*–*16*). Unlike mass-independent anomalies, mass-dependent isotope fractionations trace physical and chemical processes, such as evaporation and volatile loss. Furthermore, some of the BSE’s elemental ratios, such as Mg/Si, suggest a much more limited contribution from ECs than what would be expected based on nucleosynthetic isotope compositions alone (*14*). Instead, the BSE’s Mg and Si stable isotope compositions might be explained by the Earth accreting from large amounts of differentiated materials, which had previously undergone substantial volatile loss, such as the parent bodies of achondritic meteorites (*15*).

Nonetheless, because of analytical challenges that prevented the identification of nucleosynthetic isotope anomalies of volatile elements for decades, constraints on the origin of Earth’s volatile inventory are much more limited. These elements, which condense at relatively low temperatures of <1100 K (*17*), are fundamental for the emergence of life, with the six most abundant elements in living organisms (H, C, N, O, P, and S) being highly to moderately volatile. Yet, all Solar System materials are variably depleted in volatiles relative to the Sun and to Ivuna-like carbonaceous chondrites (CIs), a group of very volatile-rich primitive meteorites (*17*). Different extents of volatile depletion are observed in the terrestrial planets and the Moon (*18*–*21*), NCCs and achondrites range from very depleted to enriched in volatiles (*22*–*27*), and CCs are typically volatile-rich (*28*).

For many years, volatile-rich objects from the CC reservoir have thus been suggested to have supplied volatiles to the terrestrial planets (*29*, *30*). This hypothesis was substantiated when the dichotomy between the inner and outer Solar System was recently shown to extend to nucleosynthetic isotope anomalies of two moderately volatile elements, Zn and K (*5*–*7*, *31*). These studies revealed that both NC and CC reservoirs provided substantial contributions to Earth’s K and Zn inventories. However, meteorites from differentiated bodies, which have been proposed to account for most of the Earth’s mass (*32*–*34*), are still largely uncharacterized for isotope anomalies of volatile elements, such that their contribution to the Earth’s volatile budget remains poorly constrained.

## RESULTS

**Fig. 1.**
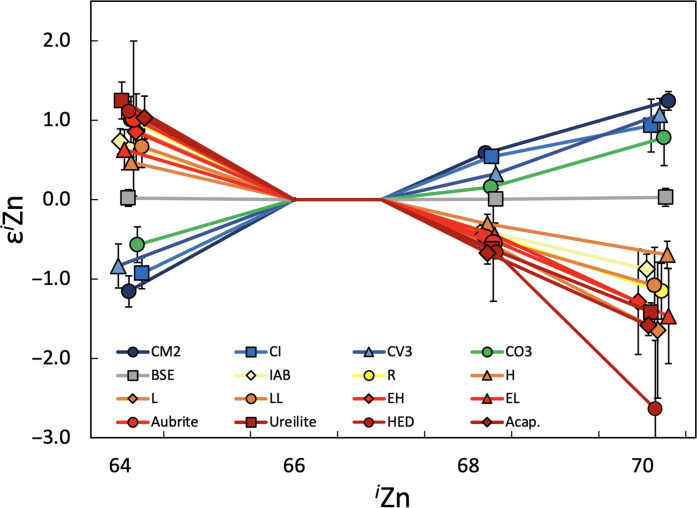
Mean Zn isotope compositions for the meteorite groups and the BSE, in ε*^i^*Zn notation. The plotted results reflect compiled data from this study and from Martins *et al.* (*7*) for CCs (including the CI, CM, CV, and CO groups) and NC group meteorites (ECs, including five EHs, one EL, OCs, including H, L, and LL chondrites, IAB complex irons, ureilites, HEDs, and acapulcoites) using the ^66^Zn/^67^Zn ratio for internal normalization. The CC and NC groups have complimentary patterns with CCs displaying negative ε^64^Zn and positive ε^68^Zn and ε^70^Zn values (relative to the BSE with ε*^i^*Zn = 0). The NCs have the opposite pattern. The terrestrial sample is indistinguishable from the Zn standard that defines εZn = 0 and intermediate between CCs and NCs. The meteorite and BSE data are listed in Table 1 and by Martins *et al.* (*7*). Uncertainties for groups with a single sample correspond to ±2 SE of that sample (EL, aubrite, acapulcoite, CO, and BSE); for groups with two samples (H, LL, and R), uncertainties correspond to ±2 SD; and for groups with more than two samples, the uncertainties correspond to ±2 SE.

The present study focuses primarily on characterizing the mass-independent Zn isotope compositions of meteorites derived from differentiated planetesimals, including both the silicate and metal fractions, as sampled by achondrites and iron meteorites, respectively, but additional CCs and NCCs are also included (see Materials and Methods). The results are reported using the ^66^Zn/^67^Zn ratio for internal normalization, but data obtained with two alternative normalization ratios are available in the Supplementary Materials. Measurements of the terrestrial rock standard BCR-2 show no substantial deviations from ε*^i^*Zn = 0 (BSE in Fig. 1). Conversely, the meteorite results show resolvable deviations from the terrestrial standard for all samples analyzed in this study (Table 1).

**Table 1.** Mass-independent Zn isotope data (in εZn notation) for the meteorites and BSE samples. *m* is the number of sample digest aliquots processed separately through column chemistry. *n* is the total number of individual analytical runs for a given meteorite, for one or several powder/digest solution aliquots.

		Data normalized to ^66^Zn/^67^Zn											
Sample		ε^64^Zn	2 SE	2 SD	*m*/*n*	ε^68^Zn	2 SE	2 SD	*m*/*n*	ε^70^Zn	2 SE	2 SD	*m*/*n*
Orgueil	CI1	−0.93	0.20	0.62	1/10	0.54	0.08	0.32	1/17	0.93	0.33	0.88	1/7
Murchison	CM2	−1.32	0.34	0.76	1/5	0.53	0.09	0.34	1/13	1.12	0.49	1.38	1/8
Winchcombe	CM2	−0.92	0.21	0.52	1/8	0.57	0.09	0.37	1/15	1.24	0.22	0.59	1/7
NWA 13299	EH3	0.76	0.24	0.80	2/11	−0.42	0.19	0.78	1/17	−1.16	0.50	1.23	1/6
MIL 07028	EH3	0.61	0.24	0.59	1/6	−0.18	0.14	0.45	1/10	−1.20	0.44	0.88	1/4
PCA 91238	EH3	0.97	0.29	0.70	1/6	−0.49	0.17	0.62	1/13	−1.07	0.51	1.34	1/7
LAR 06252	EH3	0.96	0.26	0.64	1/6	−0.49	0.08	0.27	1/12	−1.32	0.24	0.48	1/6
Indarch	EH4	0.93	0.29	0.88	1/9	−0.70	0.16	0.61	1/15	−1.67	0.67	1.63	1/6
MAC 88136	EL3	0.63	0.25	0.76	1/9	−0.45	0.15	0.54	1/13	−1.47	0.60	1.20	1/4
Tennasilm	L4	1.17	–	0.70	1/2	−0.85	–	0.70	1/2	–	–	–	–
NWA 11880	R3.5-4	1.01	0.29	0.82	2/8	−0.41	0.11	0.46	2/17	−1.15	0.35	1.05	2/9
NWA 11754	Ureilite	1.35	0.30	0.79	2/7	−0.69	0.23	0.82	2/13	−1.47	0.35	0.86	2/6
NWA 11757	Ureilite	0.97	0.21	0.52	1/6	−0.53	0.18	0.54	1/9	−1.51	1.04	2.08	1/3
NWA 11890	Ureilite	1.43	0.29	0.82	1/8	−0.65	0.10	0.39	1/16	−1.27	0.18	0.50	1/8
NWA 11395	Howardite	1.87	0.86	1.49	1/3	−1.31	0.33	0.58	1/3	−3.33	0.85	1.48	1/3
NWA 12265	Eucrite	1.13	0.84	1.89	1/5	−0.41	0.29	0.65	1/5	−1.64	0.86	1.93	1/5
NWA 14443	Eucrite	1.18	0.33	0.87	1/7	−0.58	0.18	0.48	1/7	−1.68	0.87	1.94	1/5
NWA 8287	Acapulcoite	1.03	0.27	0.85	1/10	−0.67	0.14	0.59	1/17	−1.58	0.13	0.34	1/7
Djoua	Aubrite	1.40	1.06	2.12	1/4	−0.57	0.62	1.24	1/4	−2.55	3.22	5.57	1/3
Toluca	IAB complex	0.69	0.42	0.72	1/3	−0.36	0.16	0.42	1/7	−0.67	0.74	1.48	1/4
Nantan	IAB complex	0.59	0.47	0.82	1/3	−0.48	0.11	0.31	1/8	−1.06	0.37	0.83	1/5
BCR-2	Earth	0.02	0.11	0.72	6/46	0.01	0.04	0.44	6/114	0.03	0.10	0.93	6/68

In line with other isotope systems and previous Zn results (*7*), CCs display negative ε^64^Zn and positive ε^68^Zn and ε^70^Zn values, while the NC meteorites show complementary patterns (Fig. 1 and Table 1). All NC achondrites and irons largely overlap with NCCs within uncertainties. This stands in contrast to some other Fe-peak elements as ureilites, howardite–eucrite–diogenites (HEDs), and acapulcoites are more depleted in the neutron-rich isotopes ^48^Ca, ^50^Ti, and ^54^Cr than NCCs (*1*, *3*). However, differences between NCCs and NC achondrites may become resolvable once higher-precision mass-independent Zn isotope data become available. Nonetheless, it is possible that the nucleosynthetic Zn isotope compositions of NC materials are more homogeneous compared to what has been observed for more refractory elements. Critically, in contrast to most other nucleosynthetic isotopes systems, ECs have Zn isotope compositions that are distinct from the BSE and identical to OCs.

## DISCUSSION

### Source of the anomalous Zn

**Fig. 2.**
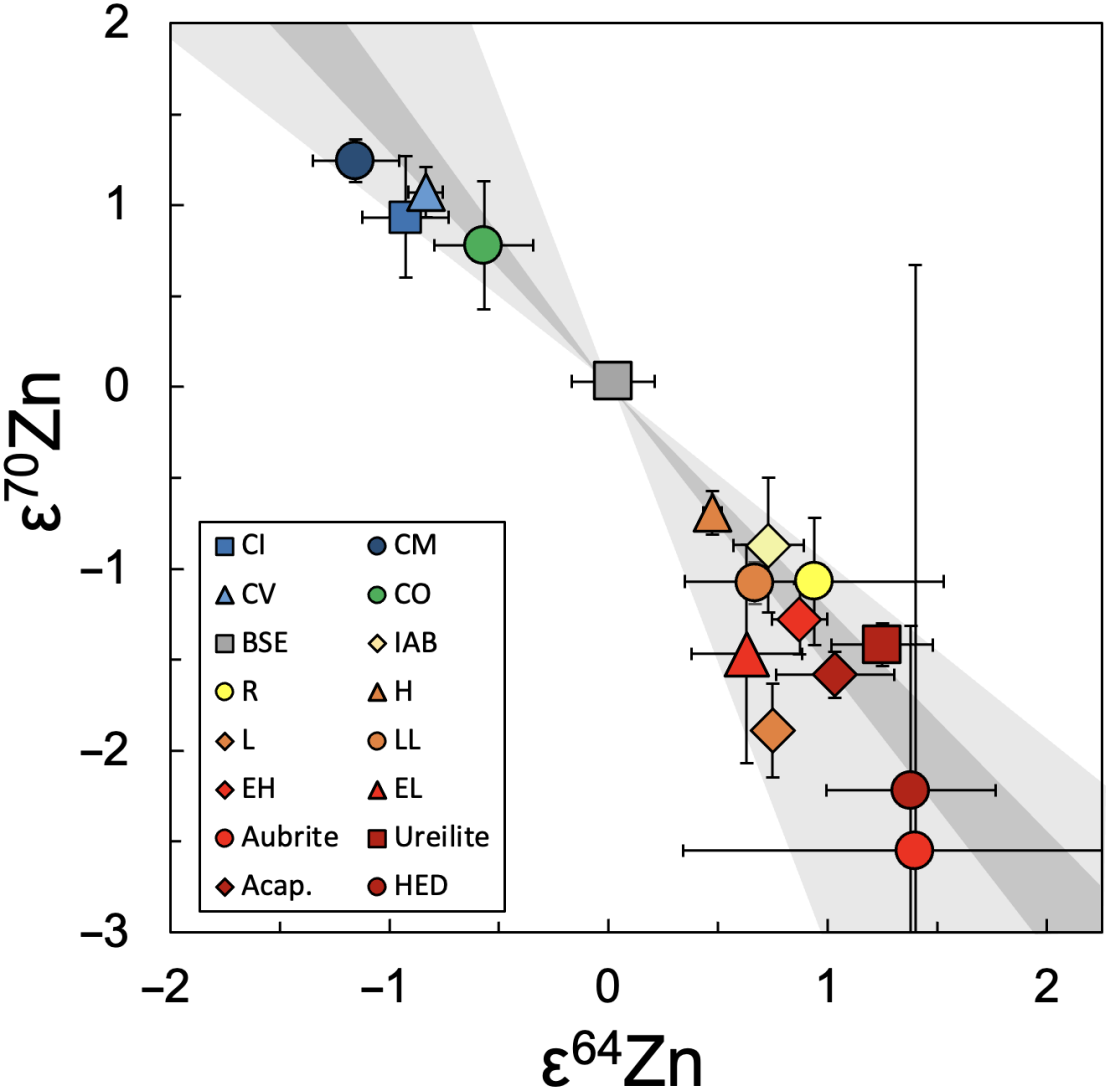
Measured Zn isotope compositions in comparison to predicted nucleosynthetic isotope anomalies. Group means encompass results from this study and from Martins *et al.* (*7*) and are shown for data obtained with internal normalization to ^66^Zn/^67^Zn. Measured isotope compositions for meteorites are in agreement with modeled anomalies for SNIa (light gray) and ECSN (dark gray).

The Zn isotope anomalies are likely produced in the same sites as the anomalies of other Fe-peak elements. Thus, the results obtained here are compared to modeled nucleosynthetic isotope anomalies produced by type Ia supernovae (SNIa) (*35*) and electron capture core-collapse supernovae (ECSN) (*36*), which have been previously linked to ^48^Ca and ^50^Ti anomalies (*1*). The models were produced following the procedures described by Martins *et al.* (*7*). The measured anomalies are generally compatible with both nucleosynthetic sources, regardless of the isotope ratio used for internal normalization (Fig. 2 and fig. S2), supporting a nucleosynthetic origin for the anomalies.

**Fig. 3.**
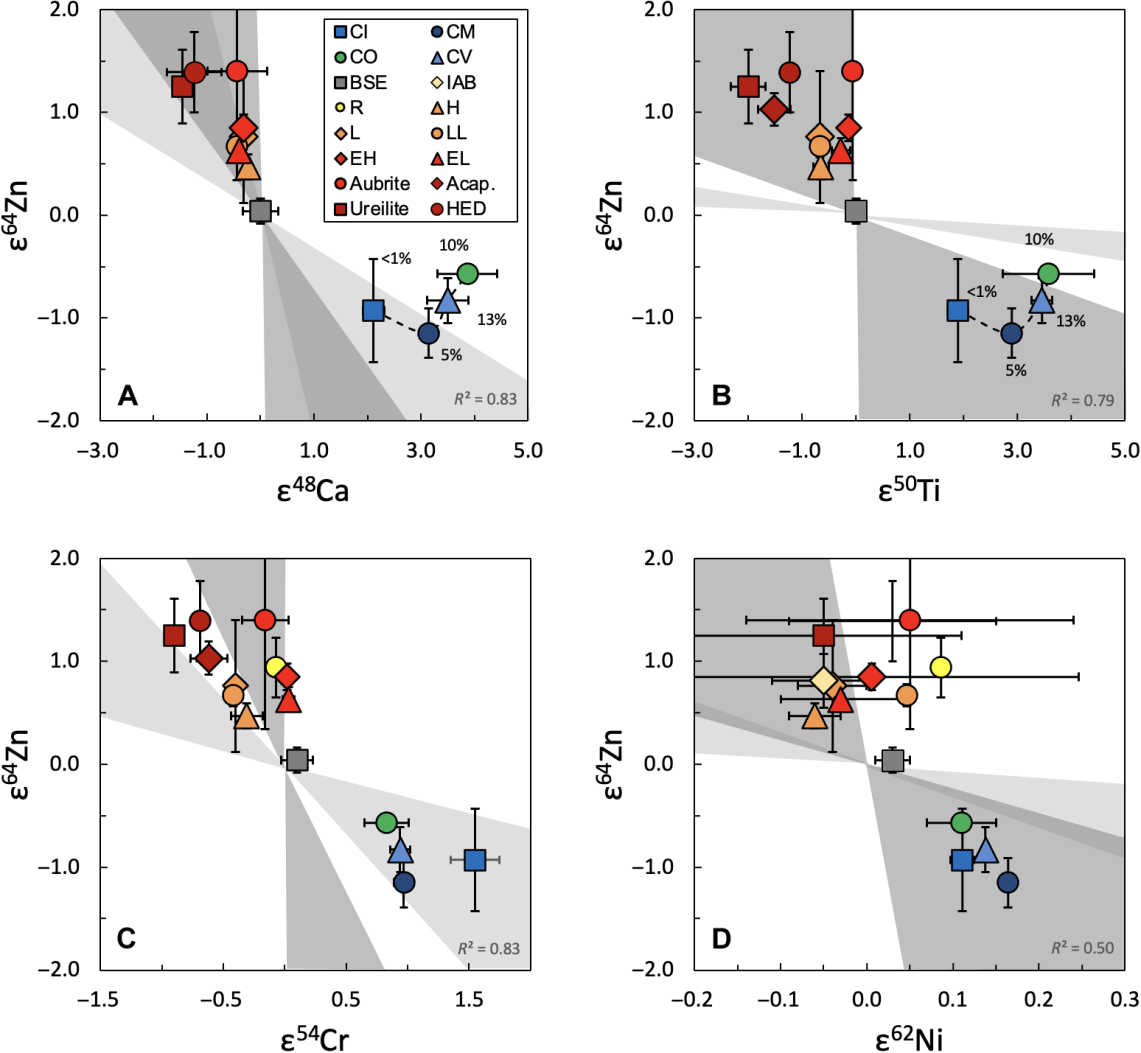
Measured ^64^Zn isotope anomalies versus isotope anomalies of other Fe-peak elements. The results are in agreement with predictions for anomalies of (**A**) ^48^Ca, (**B**) ^50^Ti, (**C**) ^54^Cr, and (**D**) ^62^Ni. The light gray and dark gray fields correspond to modeled anomalies for SNIa and ECSN sources, respectively. (A) and (B) also show the estimated volume (%) of CAIs in the CC meteorite classes. The Ca and Ti isotope anomalies roughly increase with the volume of CAIs, while the Zn isotope anomalies barely change, causing the trend to diverge from the modeled composition predicted for nucleosynthesis in SNIa and ECSN environments.

Furthermore, the Zn results correlate with measured isotope anomalies of the other Fe-peak elements (Fig. 3 and fig. S3) (*7*). These correlations do not, however, precisely align with the predicted compositions of either of the nucleosynthetic models. While NCs appear to be generally more compatible with ECSN than SNIa, the magnitude of the Zn isotope anomalies of CCs are often smaller (in terms of ε^64^Zn) than predicted by the ECSN model for a given anomaly of another element, especially Ca and Ti. This is not unexpected as the latter elements have substantially higher condensation temperatures than Zn, such that incomplete condensation of Zn from the stellar source, subsequential thermal processing, and/or different carrier phases could potentially decouple the anomalies by reduced sampling of the anomalous Zn.

Notably, the magnitude of the Ca and Ti isotope anomalies appear to increase with the volume of Ca-Al-rich inclusions (CAIs), while the Zn isotope anomalies stay roughly the same (Fig. 3 and fig. S3). These refractory inclusions are thought to be the first solids to have condensed in the Solar System, at such high temperatures (>1500 K) that would prevent condensation of volatile Zn (*17*). Furthermore, Cr and Ni anomalies have also been linked to type II supernovae (SNII) (*37*, *38*), which are unlikely to be the source of the Zn anomalies (*7*). As such, these elements could be hosted in distinct carrier phases that are decoupled from Zn prior to accretion of the CC parent bodies. These observations hence preclude us from favoring either ECSN or SNIa as a potential source of the Zn isotope anomalies based on these correlations alone.

### Origin of Earth’s Zn

While the mass fraction of the carbonaceous material accreted by Earth has been estimated previously using nucleosynthetic isotope anomalies of refractory elements, these estimates do not constrain the CC contribution to Earth’s Zn budget. This is because, unlike for refractory elements, the Zn concentrations of different Solar System materials vary substantially. In general, CCs are rich in Zn, with concentrations ranging from ~100 to almost 400 μg g^−1^ (*28*), but NCCs can have as little as a few micrograms per gram to over 300 μg g^−1^ of Zn (*22*, *39*). The Zn concentrations of achondrite parent bodies are less well constrained, with measurements of achondritic meteorites recording variable concentrations from less than 1 μg g^−1^ to over 300 μg g^−1^ (*25*, *26*). Furthermore, the bulk Earth (BE) Zn content of about 40 to 70 μg g^−1^ (*18*, *40*–*43*) is debated due to uncertainties about the partitioning of Zn into the core, and the Zn concentrations of iron meteorite parent bodies are even more difficult to constrain (*44*).

Despite potentially contributing just 10% or less to Earth’s mass (*12*, *13*, *45*–*47*), the carbonaceous material was previously estimated to account for 30 to 50% of Earth’s Zn (*5*–*7*). This study encompasses a Monte Carlo simulation of mixtures between randomly selected meteorite groups in arbitrary proportions (Materials and Methods). They include our data for previously uncharacterized chondrites [Rumuruti (R) chondrites], and achondrites (HEDs, ureilites, aubrites, and acapulcoites), as well as results for four ECs, one CM, and more precise measurements for a CI, a CM, an L, and an IAB complex iron meteorite that had been previously analyzed (*7*).

**Fig. 4.**
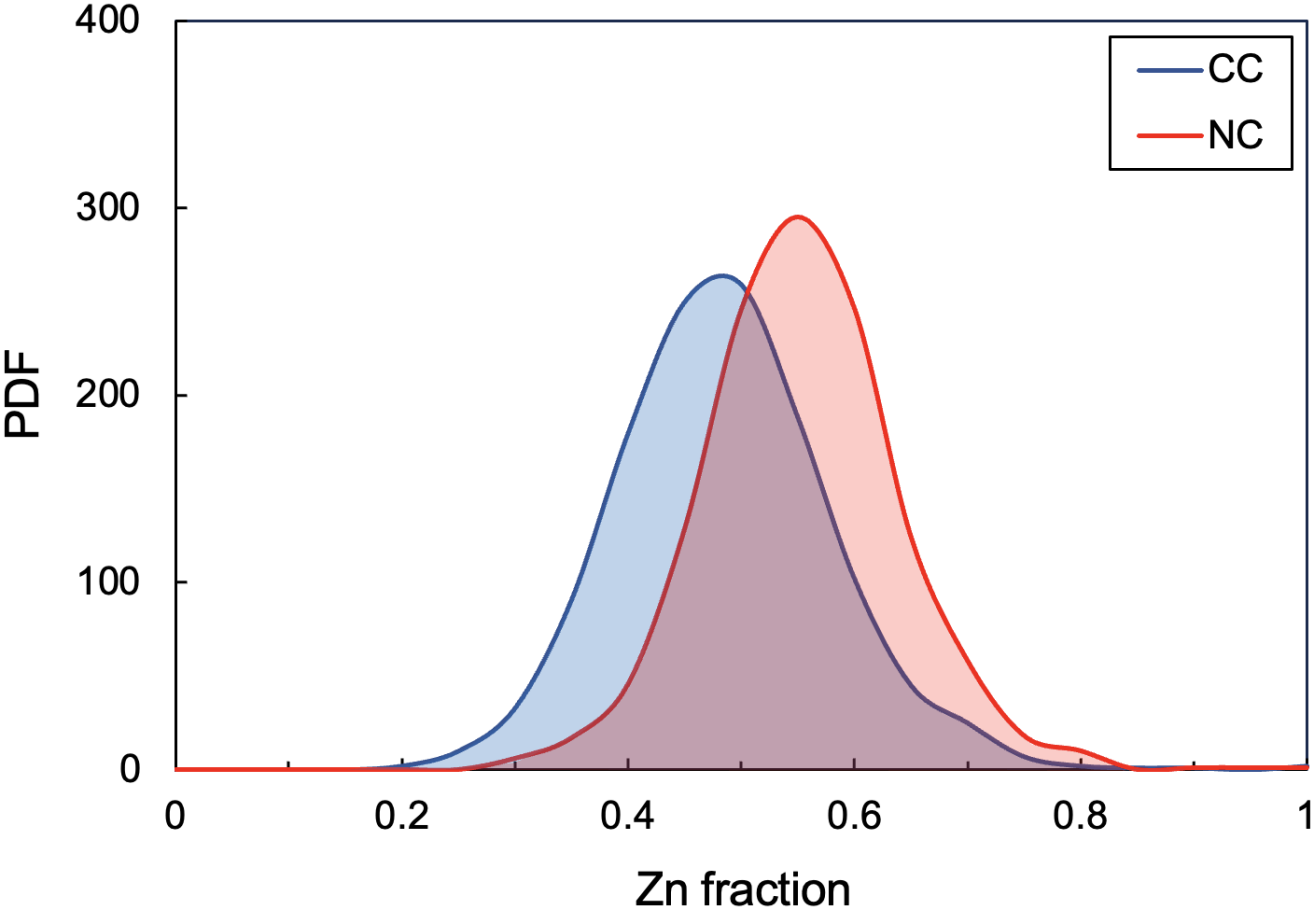
Results from simulations to determine which combinations of CC and NC meteorites successfully produce an Earth-like nucleosynthetic Zn isotope composition. The probability density functions (PDF) of the Zn mass fractions derived from CC and NC reservoirs, encompassing 1201 solutions, yield a mean ε^64^Zn = 0.03 ± 0.12 (2 SD). The average mass fraction of Zn from CCs is 46 ± 19% (2 SD), in line with estimates from Martins *et al.* (*7*). All meteorite groups characterized in this study and by Martins *et al.* (*7*) were used in the model (CI, CM, CO, CV, EH, EL, H, L, LL, R, IAB, HEDs, ureilites, aubrites, and acapulcoites)

The simulation was performed for 10,000 trials, but only results that yielded Earth-like compositions (ε^64^Zn = 0.02 ± 0.11) were selected. The 1201 valid solutions yield a mean Zn mass fraction of 46 ± 19% (2 SD) from CCs (Fig. 4), in line with the result of 48 ± 15% (2 SD) from Martins *et al.* (*7*). This is expected as the nucleosynthetic Zn isotope composition of NC achondrites does not deviate substantially from those of the NCCs used in the previous models (*7*). No CC group appears to be favored over the others, with all groups featuring in 70 to 80% of solutions and contributing an average Zn mass fraction of ~17% each. Among NCCs, R chondrites appear less frequently (39% of solutions), while OCs and ECs feature in ~75 to 90% solutions. NC achondrites also appear in ~90% of solutions, while NC irons appear in ~60%. The mean Zn fractions from NCCs, NC achondrites, and NC irons are 32 ± 14%, 19 ± 12%, and 13 ± 9%, respectively, with an overall NC mean of 53 ± 9% (2 SD).

Notably, the lower end of the range determined for the CC Zn fraction (~30 to 50%) is compatible with estimates obtained in other recent Zn isotope studies. Steller *et al.* (*6*) obtained an estimated 36 ± 19% contribution from CCs using CC and NC means, while results from Savage *et al.* (*5*) yield a range of ~20 to 45% assuming CC and EC means for the two endmembers. Furthermore, while the probability density of the Monte Carlo simulations performed in this study peaks at ~46% of Earth’s Zn being derived from CCs, other factors must be considered to assess the likelihood of this outcome, such as the availability of the materials included in the models in the desired proportions during Earth’s accretion, which is, at present, unconstrained. As such, based on the modeling results, CC contributions of anywhere between ~30 and 70% of Earth’s Zn inventory are possible.

These results contrast with an estimate for K, which suggests a contribution of just 20% from CCs (*31*). A perfect correlation between different volatile elements is, however, not to be expected because they have distinct condensation temperatures, geochemical affinities, and nucleosynthetic origins. In particular, while both Zn and K are lithophile, Zn condenses at 726 K and is produced mainly through α-rich freeze out in supernovae, while K condenses at 1006 K and is produced in SNII (*17*, *48*). Nonetheless, it is likely that CCs also played an important role in supplying other volatiles to Earth. Because the volatile depletion patterns of CCs roughly follow the sequence predicted by condensation from the solar nebula, the enrichment in Zn also extends to other moderately volatile elements with condensation temperatures of less than ~800 K—a pattern that is also observed for the BSE (*49*). Last, it is worth noting that addition of CMs would increase the estimated K contribution from CCs as these meteorites have nucleosynthetic K isotope compositions that are indistinguishable from Earth (*31*).

### The search for Earth’s accreting materials

The search for Earth’s accreting materials has led to multiple studies that used nucleosynthetic isotope anomalies as tracers to identify potential building blocks, often arriving at different mixing proportions of CC versus NC materials. An early work by Lodders (*12*) was able to reproduce the Earth’s oxygen isotope composition by mixing the 10% CI and CV material with the 90% NCC material. Albarède *et al.* (*30*) later showed that this CC mix can also be replaced by a 9% addition of CMs. Still assuming chondritic nucleosynthetic isotope compositions but using a much more comprehensive approach, Dauphas (*13*) was able to reproduce Earth’s O, Ca, Ti, Cr, Ni, Mo, Ru, and Nd isotope compositions with 5% CO-CV, 25% OC, and 70% EC material. More recently, using a similar approach but now also including Sr, Zr, Fe, Si, and Zn, these contributions were updated to <2% CO-CV and OC, 6% CI, and 92% EC (*45*).

Other studies included achondrites in their estimates, which can lead to very different results. Warren (*50*) came to a 24/76 split between CCs and NCs using Cr, Ti, and O isotope compositions for a range of chondritic and achondritic meteorites. Fitoussi *et al.* (*32*) favored addition of 18 to 28% of a CI-CV mixture to the NC material consisting of OCs and angrites. Schiller *et al.* (*33*) inferred a 42% contribution from the CI material to ureilite-like NC embryos based on Ca isotope anomalies, while Onyett *et al.* (*34*) estimated a 26% CI contribution from nucleosynthetic Si isotope anomalies, with the remaining mass coming from NC achondrites. The inclusion of achondrites in such estimates often yields larger CC mass fractions. This reflects that many NC achondrites display more extreme depletions in the neutron-rich isotopes of the Fe-peak elements than NCCs. An exception to this is the estimate of Burkhardt *et al.* (*47*), which is based primarily on s-process elements (Mo, Zr, Sr, and Nd), but also includes Cr, and which arrived at a CC contribution of just 4%.

There are, however, additional factors that favor the incorporation of achondrites, and hence differentiated planetesimals, in Earth’s building blocks. The mass-dependent Si isotope compositions of achondrites have been shown to be heavier (enriched in higher-mass isotopes) than chondrites, a feature that is also seen in the BSE (*14*). Similarly, the mass-dependent Mg isotope compositions of achondrites and the BSE are both heavier compared to results obtained for chondrites (*15*). It is, therefore, difficult to reproduce the mass-dependent isotope fractionations observed for the BSE if only chondrites are assumed as precursor materials, even when scenarios of Si sequestration into the core or a hidden Si reservoir in the lower mantle are considered (*14*). Instead, these signatures most likely reflect the accretion of differentiated planetesimals, which experienced substantial vapor loss from magma oceans during accretionary growth (*15*, *16*), resulting in the loss of isotopically light Si and Mg from these bodies.

Taking these factors into account, the models discussed in the following attempt to reproduce not only the Earth’s nucleosynthetic isotope composition but also the concentrations of key major elements. The models are based on Monte Carlo simulations in which arbitrarily selected meteorite groups were mixed in randomly generated proportions (Materials and Methods). The isotope data included in the models are Δ^17^O, ε^48^Ca, ε^50^Ti, ε^54^Cr, ε^64^Zn, ε^84^Sr, ε^96^Zr, and ε^30^Si, while the elemental data encompass the major elements Na, Mg, Si, P, Ca, K, Ti, Fe, and Mn, which were normalized to Al and to CI chondrites (table S3 and data S1). Initial tests with 10^8^ simulations revealed that the ε^96^Zr or ε^30^Si values of the BSE could be not reproduced by mixing the meteorite groups included in this study. As such, while the resulting Zr and Si mass-independent Zr and Si isotope compositions are reported, they were not used as requirements to select valid solutions. Results were further calculated for δ^30^Si and δ^25^Mg as well as the Al-normalized O, Zn, Sr, and Zr concentrations, but these data were also not used as criteria for the selection of valid solutions.

Because of the lack of data for some isotope systems (ε^96^Zr and ε^84^Sr) and bulk parent body major element concentrations, R chondrites, acapulcoites, ureilites, aubrites, and IAB complex irons were not included in the models. As such, we use only the CC, EC, and OC chondritic groups, as well as the parent bodies of eucrite and angrite achondrites (EPBs and APBs, respectively), for which bulk compositions have mostly been estimated (see Materials and Methods).

**Fig. 5.**
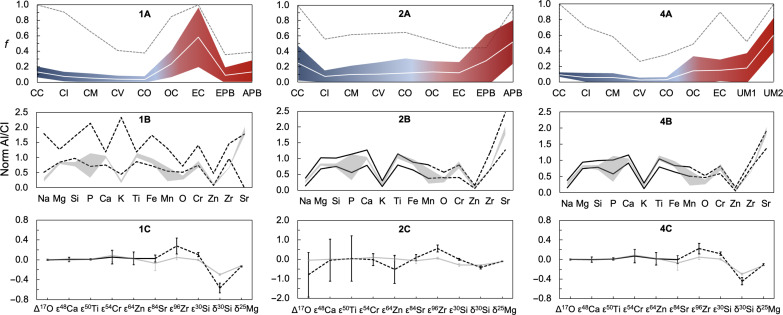
Mixing models that attempt to reproduce the isotope and elemental compositions of the BE. The numbers on the panels correspond to the Run number of the simulation (Table 2). (**A**) Mean mass fraction obtained for each meteorite group for all valid solutions (white lines) and respective uncertainties (2 SD, colored fields). The dashed lines illustrate the frequency at which each group appears in valid solutions, where *f* = 1 means that a given group appears in 100% of valid solutions. The CC fraction denotes the sum of all CC groups. (**B**) Ratio of a given element to Al, normalized to CI chondrites. The lines represent the highest and lowest values obtained for all valid solutions, while the gray fields show estimates for the BE (table S4). Elements shown with a solid line were used as criteria for the selection of valid results, while elements with dashed line were not. (**C**) Mean isotope compositions for all valid solutions of each run, with error bars indicating twice the SD. Ratios shown with a solid line were used as criteria for selection of valid results, while ratios with a dashed line were not. The gray lines denote literature data for the BSE (table S3).

The results of the models are shown in Fig. 5 and summarized in Table 2. These include the mass fractions of each meteorite group and the resulting isotope ratios and major element compositions; all of which are reported as the means and twice the SD of all valid solutions. In the first run (Run 1), which encompassed 10^9^ simulations, only the results that yielded Earth-like nucleosynthetic isotope compositions (for Δ^17^O, ε^48^Ca, ε^50^Ti, ε^54^Cr, ε^64^Zn, and ε^84^Sr) were selected. This resulted in an average *f*_CC_ mean of 13 ± 7% (2 SD), with CIs appearing most frequently (in 90% of solutions) followed by Mighei-like (CMs; 65%), Vigarano-like (CVs; 41%), and lastly Onans-like (COs; 38%) CCs, with mass fractions ranging from 4 ± 4% for COs and CVs to 7 ± 6% for CIs. As expected, ECs are by far the most recurring group, appearing in 100% of solutions with a mass fraction of 58 ± 38%, while OCs appear in 85% of solutions with a fraction of 24 ± 17%. Last, APB and EPB feature in 39 and 36% of solutions, but their mass fractions are limited to just $13_{-13}^{+15}\%$ and $9_{-9}^{+10}\%$ , respectively. The CC mass fraction is compatible with estimates by some studies that used chondrites as endmembers (*13*, *30*). As expected, however, this approach yields a BE that is too volatile-rich, with Na/Al, Mg/Al, Si/Al, and K/Al ratios that clearly exceed all current estimates. Furthermore, it also generates a BSE mass-dependent Si isotope composition (δ^30^Si = −0.57 ± 0.10) that is much lighter than current data-based estimates [δ^30^Si = −0.30 ± 0.02; data S1]. Last, the mean ε^96^Zr of all valid trials (ε^96^Zr = 0.28 ± 0.16) is incompatible with measured values (ε^96^Zr = 0.05 ± 0.05), as is the mean ε^30^Si (modeled ε^30^Si = 0.10 ± 0.04; measured ε^30^Si = −0.002 ± 0.015).

**Table 2.** Summary of results from the Monte Carlo simulations. Groups included in the models: CI, CM, CO, CV, EH, EL, H, L, LL, eucrite parent body (EPB), angrite parent body (APB), and unsampled materials 1 and 2 (UM1 and UM2: eucrite- and angrite-like elemental abundances and mass-dependent isotope compositions but OC- and EC-like nucleosynthetic isotope compositions for UM1 and UM2, respectively). Checkmarks indicate BE characteristics that are successfully reproduced in each run.

Run	1	2	3	4
Nucleosynthetic isotope composition	✓		✓	✓
Major element ratios		✓	✓	✓
Endmembers	CC, NCC, APB, EPB	CC, NCC, APB, EPB	CC, NCC, APB, EPB	CC, NCC, UM
Trials	10^9^	10^6^	10^8^	10^10^
Solutions	1461	3877	0	183
CC (%, ± 2 SD)	13 ± 7	25 ± 23	–	10 ± 3
NCC (%, ± 2 SD)	78 ± 22	15 ± 14	–	21 ± 13
APB/EPB/UM (%, ± 2 SD)	$13_{-13}^{+14}$	62 ± 17	–	70 ± 13

In Run 2, 10^6^ simulations were performed, but only the solutions that yielded elemental ratios within 20% of terrestrial values were selected. The general 20% window was allowed because concentration measurements and estimates are typically not accompanied by uncertainties. Following these criteria, the results yield a much wider range of CC mass fractions of 25 ± 23%, with all CC groups appearing in 50 to 60% of solutions and with mean fractions ranging from 8 ± 8% for CIs to $12_{-12}^{+19}$ % for COs. While ECs and OCs are still featured prominently, appearing in 44 and 45% of valid solutions, respectively, the mass fractions of these groups are much more limited in this scenario, with both averaging at $12_{-12}^{+14}\%$ and $13_{-13}^{+14}\%$ , respectively. Last, APB and EPB appear in 96 and 45% of solutions, with mean mass fractions of 52 ± 28% and $28_{-28}^{+34}$ %, respectively. Notably, even the elemental ratios that were not used as criteria for the selection of valid results are also reproduced in Run 2. This stands in contrast to Run 1, where the Sr/Al, O/Al, and Zr/Al ratios were lower than estimates for the BE. Run 2 also produced δ^25^Mg values that largely overlap with BSE estimates (δ^25^Mg = −0.121 ± 0.005), with a mean δ^25^Mg = −0.11 ± 0.02 for all valid solutions, although the Mg isotope data were not used as criteria for the selection of acceptable results. Although still lighter, the mean δ^30^Si value of all valid trials in Run 2 (δ^30^Si = −0.41 ± 0.08) is much closer to BSE estimates [δ^30^Si = −0.30 ± 0.02; data S1] than Run 1 (δ^30^Si = −0.57 ± 0.10). However, the Run 2 mixing proportions lead to highly variable nucleosynthetic isotope compositions, which are mostly incompatible with Earth.

Last, Run 3 performed 10^8^ simulations that used both the nucleosynthetic isotope compositions and elemental data as criteria for the selection of valid results. This run, however, yielded no valid solutions, which implies that a scenario in which Earth accreted only from known sampled materials is highly unlikely. Thus, it appears that a component with achondrite-like volatile element depletions but NCC-like nucleosynthetic isotope compositions is required. A potential solution for this is if aubrites are considered as one of the main contributors to Earth’s accretion. These achondritic meteorites have nucleosynthetic O, Ca, Ti, Cr, and Ni isotope compositions that are indistinguishable from Earth, and they are substantially more volatile-depleted than ECs, with Zn concentrations ranging from 0.2 to 13 μg g^−1^ (*51*). However, the nucleosynthetic isotope compositions of s-process elements and parent-body elemental concentrations for this meteorite group are unavailable, making it difficult to test this hypothesis. However, it is worth noting that the δ^30^Si value of aubrites [δ^30^Si = −0.60 ± 0.07; (*52*)] is indistinguishable from some chondrites and clearly lighter than the BSE.

### Unsampled material

The models suggest that Earth likely accreted an unsampled, volatile-depleted material with an NCC-like nucleosynthetic isotope composition. Therefore, another simulation (Run 4) was performed, which included two hypothetical unsampled materials, representing differentiated planetesimals. Because ECs and OCs accounted for ~90% of the mass in Run 1, which successfully reproduced nucleosynthetic isotope compositions, in Run 4, the unsampled materials are assumed to average out to OC- and EC-like nucleosynthetic isotope compositions, with mean OC and EC values attributed to unsampled materials 1 and 2 (UM1 and UM2), respectively (table S3). Furthermore, achondrites contribute most to Earth’s mass in Run 2 (62 ± 17%), such that their elemental abundances appear to be most compatible with the BE. In addition, the mass-dependent Si and Mg isotope compositions of achondrites are also heavier than those of chondrites and largely overlap with BSE values (table S3). Hence, the elemental abundances and mass-dependent Si and Mg isotope compositions of both UM1 and UM2 were allowed to vary within estimates for the EPB and the APB.

For Run 4, both the nucleosynthetic isotope and the major element compositions were used as criteria for selection of valid results, in addition to the final BE Zn concentration, which is estimated to be between 40 and 70 μg g^−1^ (*19*, *43*, *44*). This yielded a 10 ± 3% CC contribution (ranging from 3 ± 3% for COs and CVs to 6 ± 6% for CIs), with 21 ± 13% from NCCs ( $15_{-15}^{+19}$ % for OCs and 15 ± 14% for ECs) and the remaining 70 ± 13% coming from the unsampled materials ( $18_{-18}^{+19}$ % from UM1 and 60 ± 22% from UM2). As expected, despite not being used as criteria, the remaining elemental data (O, Cr, Zn, Zr, and Sr) are largely compatible with the BE values. The resulting δ^25^Mg values are also often consistent with the BSE (δ^25^Mg = −0.11 ± 0.02), while the mean δ^30^Si is still lighter than the BSE (δ^30^Si = −0.44 ± 0.08) but similar to the Run 2 results. As such, with the exceptions of Si and Zr, the Earth’s nucleosynthetic isotope composition and its volatile element depletion can be accounted for by accreting primarily from differentiated planetesimals with nucleosynthetic isotope compositions akin to a mix of OCs and ECs.

The need for some unsampled materials in Earth’s accretion was previously raised because the nucleosynthetic Mo isotope composition of the BSE requires the addition of a material that is more enriched in s-process nuclides than any known meteorites (*53*). Furthermore, the Zr isotope composition of the valid trials of Run 4 (ε^96^Zr = 0.22 ± 0.11) are also s-process depleted compared to measured BSE values (ε^96^Zr = 0.05 ± 0.05; data S1), suggesting that addition of a distinct s-process enriched material is also recorded by the Zr isotope composition of the BSE (table S3). It is, therefore, likely that at least some of the unsampled material proposed in this study is more enriched in s-process isotopes than the EC or OC compositions that currently define UM1 and UM2. This would satisfy the Mo and Zr isotope constraints and possibly change the proportions of CC versus NC materials.

However, the effect of this distinct s-process enriched material is much more prominent for the BSE’s Mo isotope composition than for Zr isotopes. In detail, while ~50% of the Mo in the BSE appears to have been supplied by the s-process enriched material (*47*, *54*), the Run 1 and 4 models already yield ε^96^Zr estimates that are nearly in accord with the BSE value, considering the combined uncertainties (Fig. 5). The ε^96^Zr data hence imply more limited contributions of the s-process enriched material to the BSE than the Mo-based estimates. Furthermore, the model results for ε^84^Sr, which also records the addition of s-process nuclides, largely overlap with the measured BSE values for all three runs, showing that the distinct s-process enriched material is not required to reproduce the Sr isotope BSE value, given the current precision of the data.

The different enrichment levels of s-process nuclides inferred from Mo versus Zr and Sr isotopes likely reflect the different metal-silicate partitioning of these elements. In detail, early accreted Mo was largely segregated into the core during differentiation due to the siderophile nature of the element, whereas lithophile Zr and Sr preferentially stayed in the BSE. The BSE’s Mo isotope composition thus reflects the last ~10 to 20% of Earth’s accreting materials, while Zr and Sr record the compositions of most of the Earth’s accreting materials (*13*). As the isotope systems used in our models were all primarily lithophile during Earth’s accretion, the timing of the UM1 and UM2 additions is not constrained by the models. It is, therefore, possible that not all of the UM1 and UM2 additions were s-process enriched, but that this enrichment was only imparted by about half of the final 10 to 20% of the accreted material (~5 to 10%). This would have a major impact on the BSE’s Mo isotope composition while inducing much smaller changes on the s-process enrichments of Zr and Sr isotopes. Hence, while the mass contribution of the distinct s-process enriched material is not sufficiently constrained to enable meaningful estimates of its nucleosynthetic Zr and Sr isotope compositions, it was likely substantially smaller than the total proportion of the accreted UM1 and UM2 material (~70%), and this limits its impact on the overall modeling results (Fig. 5).

Given that the timing of accretion of the different materials is not constrained by the present models, late addition of both CC and unsampled s-process enriched NC materials can be accommodated, in line with the BSE’s Mo isotope composition (*47*). Alternatively, it has been suggested that the unsampled s-process enriched component may be of CC origin (*54*). In this scenario, the BSE is inferred to record late addition of this unsampled CC component combined with a similar proportion of the EC material. However, the unsampled material would still display CC-like isotope compositions for Fe-peak elements, such that this would not affect their mass balance in the models. Hence, ~10 to 20% late accretion comprising a mix of EC and the s-process enriched CC material is also compatible with the model results, which imply the addition of the ~10% CC and ~15% EC material throughout Earth’s accretion history. Thus, while it is possible that some of the proposed unsampled and differentiated NC material accounts for the BSE’s s-process enrichment, the prospect that another unsampled component (s-process enriched CC material) was accreted late cannot be rejected based on the present results.

Last, the ε^30^Si values produced in Run 4 are also incompatible with BE estimates (*34*). This is because, unlike for other isotope systems, the Earth’s mass-independent Si isotope composition is intermediate between those of chondrites and achondrites, such that it is impossible to reproduce by mixing of chondrites alone. Thus, if the ε^30^Si results were used as a requirement in the models, then Runs 1 and 4 would have yielded no results. However, Dauphas *et al.* (*45*) posited that the results reported by Onyett *et al.* (*34*) may partially reflect mass-dependent isotope fractionation. This is particularly plausible for ECs as these meteorites display very fractionated δ^30^Si values relative to the BSE (*52*), so that the applied corrections are possibly inadequate. If so, then the mass-independent Si isotope results would not impede a chondritic model.

Alternatively, if the mass-independent Si isotope compositions reported by Onyett *et al.* (*34*) can be entirely attributed to nucleosynthetic anomalies, then the mixing proportions between CC and NC materials proposed in that study are still incompatible with other isotope systems and elemental abundances. Run 4 of our models suggests a 30/70 split between the chondritic and achondritic material, which is similar to the proportion (26/74) proposed by Onyett *et al.* (*34*). The latter estimate, however, considers only CI-like dust as the chondritic endmember, while Run 4 suggests that the 30% of the chondritic material is split between CCs (~10%) and NCCs (~20%); both of which have markedly lower ε^30^Si values than CIs (table S3). For the mass-independent Si isotope results of Run 4 to agree with the BSE value using a chondritic component that is a mix of CC and NCC materials, this requires that both UM1 and UM2 have ε^30^Si values that are intermediate between those of the BSE and achondrites. The latter, however, are clearly distinct from the EC and OC compositions ascribed to them for all other nucleosynthetic isotope systems and from any other materials analyzed in that study (*34*).

Notably, the ε^30^Si-based CI contribution estimate of 26 ± 9% (*34*) assumes invariable Si concentrations for the accreting materials, contrary to the distinct measured and estimated values for the endmembers included in the estimate (CIs and NC achondrites) and the BE (table S4). All chondrite groups display similarly light δ^30^Si values (table S3), which likely reflect equilibrium condensation of different chondrite components from a nebular gas (*55*), while the heavier δ^30^Si values of the BSE and bulk achondrite parent bodies have been linked to evaporative loss (*15*, *16*) or silicate-metal fractionation during core formation (*56*). Hence, Si loss from at least some of the accreting materials is likely required to reproduce the BSE’s mass-dependent Si isotope composition, and this would also affect the ε^30^Si mass balance. To account for such discrepancies, Onyett *et al.* (*34*) proposed that thermal processing of the pebble material during its passage through the gas envelopes around planetary embryos could not only account for volatile loss but also decouple nucleosynthetic isotope anomalies, due to the distinct volatilities and thermal stabilities of different elements and their carrier phases. Such thermal processing is, however, very poorly constrained for carrier phases other than SiC and for elements other than Mo. Further studies are hence required to test if this mechanism is compatible with the observed BE isotope and elemental abundances. The possibility that Si also records the accretion of some unsampled materials cannot therefore be discarded at present.

### Accretion and volatile depletion

The models presented in this study constrain not only the origin (inner or outer Solar System) but also the extent of volatile depletion of Earth’s precursor materials. In detail, the average Zn concentrations of the materials that provided valid results in Run 4 were calculated, yielding 52 μg g^−1^ for the BE, 244 μg g^−1^ for CCs, 52 μg g^−1^ for OCs, 178 μg g^−1^ for ECs, and 11 μg g^−1^ for the unsampled materials. This implies that, despite providing most of the Earth’s mass (~70%), the unsampled differentiated materials only contributed ~10% of Earth’s Zn inventory, with the remaining Zn fraction supplied by a mix of undifferentiated CCs and NCCs. Consequently, undifferentiated materials were a fundamental source of Zn and likely other volatiles for the terrestrial planets. This is consistent with the more limited extents of volatile depletion experienced by the different chondritic materials. While processes in the protoplanetary disk, and to some extent on parent bodies, imparted variable volatile depletions on chondritic meteorites (*57*–*60*), differentiated planetesimals record extensive volatile loss through degassing during melting and differentiation (*15*).

In detail, Hin *et al.* (*15*) showed that collisions alone produce enough energy to induce the formation of magma oceans on planetesimals and cause subsequent vapor loss of up to 36% of mass in bodies smaller than 0.1 M_⊕_. This mechanism for volatile loss is preferred because the heavy mass-dependent Si and Mg isotope compositions of the BSE are difficult to reconcile with either partial condensation from the solar nebula or with isotope fractionation during metal-silicate partitioning (*15*, *16*). The study of Hin *et al.* (*15*) also modeled isotope fractionation and elemental loss in such a scenario and was able to reproduce Earth’s Mg, Si, and Fe isotope compositions and concentrations. Furthermore, the decay of extinct radionuclides can also provide sufficient energy to melt early formed planetesimals that were large enough (>4 km), leading to the formation of magma oceans and subsequent evaporation of volatiles (*61*).

Yet, this extensive loss of volatiles during accretion is halted once a differentiated body has enough mass to prevent gaseous molecules from escaping its atmosphere. By comparing the velocities of molecules for an ideal gas under magma ocean conditions with the respective escape velocities, one can estimate the radii required for a planetesimal to retain its volatiles during degassing (*62*). Assuming a density of 2 to 4 g cm^−3^, which is within the range of asteroids (*63*), the minimum radius required to retain gaseous Zn is about 550 to 780 km. For heavier moderately volatile elements, such as Cd_(g)_ or Te_(g)_, the threshold radii are similar at about 400 to 600 km. Thus, until planetesimals achieve radii exceeding several hundreds of kilometers, they can be extensively devolatilized by magma ocean degassing, either as a consequence of collisions or radioactive decay. The modeling results of this study thus suggest that Earth accreted mainly (~70% of its mass) from small planetesimals that underwent substantial volatile loss, and once these planetesimals reached the critical size that prevented further volatile loss, further additions of chondritic material likely provided most of the volatile inventory.

### Implications for the formation of habitable planets

The limited Zn contributions from differentiated planetesimals is in line both with geochemical evidence from achondrites (*62*) and astrophysical modeling (*64*), which suggest that water is efficiently lost from early formed, differentiated planetesimals, likely due to the presence of ^26^Al. Contrary to earlier assumptions, it is improbable that the ^26^Al present in the early Solar System was derived exclusively from a single supernova explosion (*65*). This conclusion is important because it relaxes the constraint that newly formed planetary systems can only incorporate live ^26^Al if they form just a few million years after a stellar explosion, due to the short half-life of ^26^Al [0.7 million years (Ma)]. Instead, pre-supernova mass loss from giant stars may contribute as much as 50% to the galactic ^26^Al inventory. Such processes can continually replenish the interstellar medium over millions of years (*66*) to generate appreciable levels of ^26^Al, particularly in the high-mass star forming regions where most planetary systems are established.

Differentiation was ubiquitous among the first bodies to form in the Solar System due to the high abundance of ^26^Al (*61*) and was likely accompanied by substantial volatile loss (*15*). In contrast, chondrules formed later (1 to 4 Ma), and the parent bodies of chondritic meteorites therefore likely incorporated less ^26^Al, preventing them from melting and losing appreciable amounts volatiles. Consequently, delayed addition of undifferentiated materials to bodies that were large enough (with diameters of hundreds of kilometers) to retain volatiles during accretion may have been required to establish the volatile budget of the terrestrial planets. Because similar levels of ^26^Al are likely present in other nascent planetary systems, early formed planetesimals from planetary systems outside of our own are also expected to undergo differentiation accompanied by volatile loss. Delayed accretion of primitive asteroids may, therefore, also be required to establish the volatile budgets that are necessary for the emergence of life on Earth-like exoplanets.

While materials from the inner Solar System supplied a substantial fraction of the Earth’s Zn budget, the addition of primitive materials that formed at much greater heliocentric distances (CCs) is still required to reproduce the Earth’s nucleosynthetic Zn isotope composition. This finding also extends to K, the only other volatile element for which nucleosynthetic anomalies have been identified to date (*31*). The BSE’s nucleosynthetic K isotope composition can be reproduced by the addition of 10 to 20% K derived from CIs, COs, and/or CVs, but as the nucleosynthetic K isotope composition of CMs is indistinguishable from the BSE, even larger contributions from CCs can be accommodated. In addition, CCs and the BSE have the same volatile element depletion patterns (*49*), in line with the large accreted CC mass fraction (of 25 ± 23%) that is inferred by the Run 2 result, which attempt to reproduce the BE elemental abundances. The accretion of CCs therefore had a profound impact on Earth’s volatile inventory.

Limited mixing between the CC and NC reservoirs has been linked to the scattering of planetesimals and planetary embryos induced by orbital instabilities of the gas giants in the early Solar System (*67*). The inward migration of pebble-sized CI material during accretion of the proto-Sun has been proposed as an alternative mechanism for the growth of planetary embryos and can also account for such mixing (*33*). The mechanism by which the carbonaceous material from beyond Jupiter was transferred to the inner Solar System thus remains unclear. An important step toward addressing this uncertainty would be to better constrain the time period during which the CC material was added to Earth and determine if the material was accreted continually or in isolated events. Evidence from Ru, Ag, and Se isotopes and S, Se, and Te ratios in the BSE suggest that at least a fraction of the CC material was added late (*11*, *60*, *68*–*70*). However, the Mo isotope evidence for the proportion of the CC material that was added after core formation ceased is ambiguous (*47*, *54*). Future nucleosynthetic isotope studies of elements that are both volatile and siderophile are thus required to constrain the proportion of the CC material that was accreted late by Earth and its volatile contribution. Despite of these uncertainties, the available data already suggest that an influx of the material derived from the outer, colder regions of a given planetary system may play an important role in establishing the volatile inventory of Earth-like exoplanets.

## MATERIALS AND METHODS

### Samples and sample preparation

The study encompassed (i) eight achondrites, including three ureilites, one howardite, two eucrites, one acapulcoite, and one aubrite; (ii) two IAB complex iron meteorites; (iii) three CCs; (iv) six ECs; (v) one OC; and (vi) one R chondrite. Together, 21 meteorites were analyzed, with all relevant information, including meteorite sources, summarized in table S1. Multiple aliquots of the US Geological Survey (USGS) geological reference material BCR-2 were analyzed for quality control.

These samples were either used up completely by destructive analysis during the course of the study (Orgueil, Murchison, NWA 13299, MIL 07028, MAC 88136, Tennasilm, Djoua, Nantan, and Toluca) or remaining sample aliquots (Winchcombe, Indarch, PCA 91238, LAR 06025, NWA 11880, NWA 11754, NWA 11757, NWA 11890, NWA 11395, NWA 12265, NWA 8287, and BCR-2) are stored at the Department of Earth Science & Engineering, Imperial College London.

Digestion and preparation of the samples and the subsequent Zn isotopic measurements were carried out in the Mass Spectrometry and Isotope Geochemistry (MAGIC) Laboratories at the Department of Earth Science & Engineering of Imperial College London following the procedures outlined by Martins *et al.* (*7*). All sample preparation was conducted in International Standards Organization (ISO) Class 6 clean rooms, using ISO Class 4 laminar flow benches for critical steps. The water used was of ≥18.2 megohm·cm quality from a Millipore purification system. All acids were purified from reagent grade stock acids by sub-boiling distillation in either quartz glass (15.3 M HNO_3_ and 6 M HCl) or Teflon (28 M HF, 12 M HCl, and 8.5 M HBr) stills.

The original silicate meteorites, weighing between 0.36 and 18.2 g, were crushed in an agate mortar and pestle. Smaller fractions, ranging from 0.158 to 2.74 g, were subsequently aliquoted for this study, depending on the expected Zn content. They (and BCR-2) were then digested in Savillex Teflon beakers as follows. First, they were refluxed in a 2 + 1 mixture of 28 M HF + 15.3 M HNO_3_ at 120°C for at least 2 days on a hotplate and dried down. This process was then repeated with 6 M HCl. The digestion procedure for the iron meteorites is described in detail by Martins *et al.* (*7*). Following digestion, the samples were purified using the three-stage anion exchange procedure described by Martins *et al.* (*7*). Some samples (Orgueil, Murchison, and Toluca) were previously crushed, digested, and treated (*7*), and the results presented in this study reflect new isotope measurements.

### Isotope measurements

The isotope analyses were conducted with a Nu Instruments Nu Plasma II multiple collector inductively coupled plasma mass spectrometer. A Nu Instruments DSN 100 desolvation system fitted with glass cross flow nebulizers with solution flow rates of about 120 μl min^−1^ was used for sample introduction in conjunction with a CETAC ASX-112FR autosampler. The analyses were carried out using two different Faraday cup configurations: The M64 configuration covers the isotopes ^64^Zn, ^66^Zn, ^67^Zn, ^68^Zn, and ^62^Ni, while the M70 configuration covers ^66^Zn, ^67^Zn, ^68^Zn, ^70^Zn, and ^72^Ge (table S5). All ion beams were monitored using Faraday cups fitted with 10^11^-ohm resistors, with each run encompassing three blocks with 20 data acquisition cycles of 8 s each. Each run was started by a peak centering routine, while each block commenced with a 60-s measurement of the electronic baselines of the Faraday collectors while the ion beam was deflected in the electrostatic analyzer.

Monitoring of ^73^Ge on the H10 Faraday cup during M70 block measurements (table S5) revealed that Ge was consistently below the detection level. As such, no Ge corrections were needed, and the ion beam collected in Faraday cup H8 of the M64 block analyses was attributed exclusively to ^70^Zn. Hence, provided that the H8 beam was sufficiently high (typically >0.4 V) and stable, ε^70^Zn values collected in M64 block measurements are reported for the samples NWA 11395, NWA 12265, NWA 14443, and Djoua.

The solutions were introduced in 0.1 M HNO_3_, typically containing 400 to 500 ng g^−1^ of Zn, with instrumental sensitivity ranging between 150 and 200 V (μg ml^−1^)^−1^. All analyses were carried out with the sample-standard bracketing technique, in which sample runs were symmetrically bracketed by runs of the London Zn isotope reference material. Sample concentrations typically matched the standard concentrations within 10 to 15%.

The Zn results are reported using the ε notation, which denotes deviations of the measured isotope ratio for a sample (sam) from the value determined for the London Zn reference standard (std) in parts per 10^4^

εi/jZnk/j=Zni/ZnjsamZni/Znjstd−1×104 (1)

where *i*/*j* is the isotope ratio of interest and *k*/*j* is the normalization ratio. As data obtained via the ^66^Zn/^67^Zn normalization scheme are most frequently quoted in the manuscript, a simplified ε*^i^*Zn notation is adopted in this case. The full ε^*i*/*j*^Zn_*k*/*j*_ notation, however, is used to denote Zn isotope data normalized to ^64^Zn/^67^Zn and ^64^Zn/^68^Zn. The εZn values were calculated relative to the mean isotopic ratios of about 20 to 40 runs of the London Zn standard during the same measurement session.

### Zinc mass balance

A 10,000-trial Monte Carlo simulation of a mixture between all meteorite groups characterized here and by Martins *et al.* (*7*) was performed using the following equations

$$\varepsilon^{64}{\text{Zn}}_{\text{BE}}=\sum_{i=1}^{n}p_{i}\times \varepsilon^{64}{\text{Zn}}_{i}\times f_{i}$$ (2)

fi=ri∑i=1n(pi×ri) (3)

where *i* corresponds to 1 of the 16 different groups. A value of either 0 or 1 was randomly assigned to *p_i_* to determine if each group would be included in a given trial. The mass fraction of each group, *f_i_*, was attributed by randomly generating a number *r_i_* between 1 and 99 and then normalizing it to the sum of the *r_i_* values for all groups included in a trial (Eq. 3). The ε^64^Zn value was preferred due to the abundance of ^64^Zn isotope, which leads to better precision. The isotope compositions of each group were generated in a normal distribution, varying from the group mean within the respective uncertainty. Because Zn was, at most, only slightly siderophile during Earth’s accretion, it is reasonable to assume that ε^64^Zn_BE_ = ε^64^Zn_BSE_, which is characterized by the terrestrial sample BCR-2, with a mean of ε^64^Zn = 0.02 ± 0.11 (2 SE). The sums of the fractions of all CC and NC groups are taken to represent the Zn mass fraction derived from each reservoir, but only trials that yielded results that were in accord with the terrestrial values were considered valid.

### Multielement mass balances

The Monte Carlo simulations were designed according to the following equations

$$R_{\text{BE}}=\frac{\sum_{i=1}^{n}p_{i}\times R_{i}\times {\left[E\right]}_{i}\times f_{i}}{\sum_{i=1}^{n}p_{i}\times {\left[E\right]}_{i}\times f_{i}}$$ (4)

$${\left[E\right]}_{\text{BE}}=\sum_{i=1}^{n}p_{i}\times {\left[E\right]}_{i}\times f_{i}$$ (5)

where *i* corresponds to the different meteorite groups included in the model (tables S3 and S4). A value of either 0 or 1 was randomly assigned to *p_i_* to define if each group would be included in a given trial. The mass fraction of each group, *f_i_*, was defined by randomly generating a number *r_i_* between 1 and 20 for CCs and 1 and 99 for NCs and then normalizing it to the sum of the *r_i_* values for all groups included in each trial, according to Eq. 3. The CC mass fraction was purposefully assigned smaller values compared to NCs to optimize the number of valid solutions as most previous studies suggest that the CC mass fraction is relatively limited (*7*, *12*, *13*, *47*). Notably, *r* values for NCs were also allowed within the CC range (1 to 20), such that large proportions (>50%) of the CC material were still possible but less likely. The isotope ratios *R_i_* were generated in a normal distribution, varying within uncertainties from their respective group means (table S3). The concentrations of the element [*E*]*_i_* were randomly generated numbers between the lowest and highest measured results in the case of chondrites or estimated values for the different bulk achondrite parent bodies (table S4).

During core formation, siderophile elements (Mo, Ru, Ni, and Fe) variably partition into the Earth’s core, such that the nucleosynthetic isotope composition of the BSE for these elements is biased toward the compositions of materials that were added relatively late during Earth’s accretion. As such, the models presented here focus on lithophile elements, which trace most of Earth’s accretion. Neodymium was not included, however, because the BSE’s Nd isotope composition was possibly altered by collisional erosion and preferential loss of more incompatible elements (*71*). Likewise, the models do not take ^40^K anomalies into account as the available results are presently limited to chondritic meteorites.

Notably, estimates for the bulk Zn and Zr concentrations of the APB and EPB are not available, as is the nucleosynthetic Zn isotope composition of the APB. The Zn concentrations of the APB and EPB were thus allowed to vary within the observed ranges of the respective meteorites, which correspond to 0.11 to 8.9 μg g^−1^ and 5 to 12 μg g^−1^ for HEDs and angrites, respectively (table S4) (*26*, *27*, *72*). It is possible that the available samples are not representative of the bulk parent bodies because they may have experienced further episodes of volatile depletion, possibly due to magmatic processes or volatilization caused by impacts. However, the low Zn concentrations of diogenites [0.29 to 3.10 μg g^−1^; (*26*)], which sample deeper layers of the HED parent body, and the heavy mass-dependent Zn isotope compositions of unbrecciated eucrites imply that evaporation and Zn loss were not limited to impact-driven volatilization or crustal samples (*26*). Furthermore, as Zn does not partition strongly into a metal, the Zn concentrations of the bulk parent bodies are likely lower compared to the silicate fractions sampled by the meteorites. Hence, while the limited constraints preclude further estimates for the bulk Zn concentrations of these achondrite parent bodies, it seems unlikely that they would be substantially higher than the measured values of achondritic meteorites. As Zr concentrations do not vary as substantially as those of Zn among different Solar System materials, the Zr concentrations assumed for the APB and EPB were allowed to vary within the range of Zr abundances estimated for the BE (table S4).

Because angrites are rare (only 20 specimens) and have very low Zn contents, it was not possible to characterize the nucleosynthetic Zn isotope composition of this meteorite group for this study. However, the ε^64^Zn value for angrites was estimated independently through bivariate regressions following the method of Wu and Yu (*73*). Three regressions were performed based on the correlations between ε^64^Zn and ε^48^Ca, ε^50^Ti, and ε^54^Cr (Fig. 3), which show good to excellent agreement (*R*^2^ = 0.83, 0.79, and 0.83 for correlations between ε^64^Zn and ε^48^Ca, ε^50^Ti, and ε^54^Cr, respectively). The results of the three regressions (table S6) were averaged, and the uncertainties of the slopes, the intercepts, and the measured ε^48^Ca, ε^50^Ti, and ε^54^Cr values for angrites were propagated, resulting in an estimated ε^64^Zn = 0.89 ± 0.42 for angrites.

## Supplementary Materials

### The PDF file includes:

Tables S1 to S6 Figs. S1 to S3 Legend for data S1 References
