# Supplementary material for: Primitive asteroids as a major source of terrestrial volatiles
Source: Sci Adv. 2024 Oct 11;10(41):eado4121. doi: 10.1126/sciadv.ado4121 (PMC11468921; doi:10.1126/sciadv.ado4121)
Supplement: Supplementary file 1 — Tables S1 to S6 Figs. S1 to S3 Legend for data S1 References [file sciadv.ado4121_sm.pdf]

Science Advances
AAAS

Supplementary Materials for

Primitive asteroids as a major source of terrestrial volatiles

Rayssa Martins *et al.*

Corresponding author: Rayssa Martins, rm2185@cam.ac.uk

Sci. Adv. **10**, eado4121 (2024)
DOI: 10.1126/sciadv.ado4121

The PDF file includes:

Tables S1 to S6
Figs. S1 to S3
Legend for data S1
References

Other Supplementary Material for this manuscript includes the following:

Data S1

Table S1. Names, digested masses, identifiers and sources of the meteorites and BSE rock analyzed in this study.

Samples	Type	Total mass (g)	Digested aliquots (g)	Status	Identifier	Source
Meteorites						
Orgueil	CI1	0.36	0.158	Fully destroyed	BM.36104	Natural History Museum London
Murchison	CM2	1.26	0.612	Fully destroyed	ME 2644, #26.55	Field Museum Chicago
Winchcombe	CM2	1.9398	0.250	Stored	Bag 10.2 Stone 1	Natural History Museum London
NWA 13299	EH3	0.456	0.456	Fully destroyed		Private collector
Indarch	EH4	1.37	1.302	Stored	M4 1403, #22	Field Museum Chicago
MIL 07028	EH3	7.65	0.602	Fully destroyed	MIL 07028,33	US Antarctic Meteorite Program $
PCA 91238	EH3	0.853	0.603	Stored	PCA 91238,29	US Antarctic Meteorite Program $
LAR 06252	EH3	7.31	0.607	Stored	LAR 06252,45	US Antarctic Meteorite Program $
MAC 88136	EL3	0.54	0.761	Stored	MAC 88136,119	US Antarctic Meteorite Program $
Tennasilm	L4	2.00	2.00	Fully destroyed	USNM 483 2	Smithsonian National Museum
NWA 11880	R3.5-4	1.24	0.308	Stored		Private collector
NWA 11754	Ureilite	1.630	0.250	Stored		Private collector
NWA 11757	Ureilite	4.28	2.000	Stored		Private collector
NWA 11890	Ureilite	1.35	0.400	Stored		Private collector
NWA 11395	Howardite	11.55	2.45	Stored		Private collector
NWA 12265	Eucrite	18.2	3.5	Stored		Private collector
NWA 14443	Eucrite	2.74	2.74	Fully destroyed		Private collector
NWA 8287	Acapulcoite	0.54	0.38	Stored		Private collector
Djoua	Aubrite	2.3	2.3	Fully destroyed		Private collector
Nantan	IAB complex	7.6	7.6	Fully destroyed		Private collector
Toluca*	IAB complex	10.8	10.8	Fully destroyed		Private collector
BSE rock						
BCR-2	Columbia River Basalt		1.5	Stored		US Geological Survey

Total mass corresponds to original mass of the sample, a fraction of which was crushed to powder (typically >1g) and a powder aliquot was digested. The final digestion aliquot that was separated for this study corresponds to 10-100% of the total digested powder, depending on the mass. * Sample Toluca 2 is from Bridgestock et al. (*44*). $ US Antarctic meteorite samples are from the Antarctic Search for Meteorites (ANSMET) program funded by NSF and NASA; they are characterized and curated by the Dept. of Mineral Sciences of the Smithsonian Institution and the Astromaterials Curation Office at NASA Johnson Space Center.

Table S2. Mass-independent Zn isotope data (in εZn notation) for the meteorites and BSE samples. m: number of sample digest aliquots processed separately through column chemistry. n is the total number of individual analytical runs for a given meteorite, for one or several powder/digest solution aliquots.

		Data normalized to $^{64}Zn/^{67}Zn$							
**Sample**		**$\varepsilon^{66/67}Zn$**	**2se**	**2sd**	**m/n**	**$\varepsilon^{68/67}Zn$**	**2se**	**2sd**	**m/n**
Orgueil	CI1	0.30	0.06	0.21	1/10	0.18	0.10	0.30	1/10
Murchison	CM2	0.43	0.11	0.25	1/5	0.19	0.12	0.27	1/5
Winchcombe	CM2	0.30	0.07	0.20	1/8	0.21	0.13	0.37	1/8
NWA 13299	EH3	-0.26	0.07	0.23	2/11	-0.18	0.22	0.72	2/11
MIL 07028	EH3	-0.20	0.09	0.19	1/6	0.16	0.19	0.47	1/6
PCA 91238	EH3	-0.32	0.09	0.23	1/6	-0.32	0.26	0.65	1/6
LAR 06252	EH3	-0.32	0.09	0.21	1/6	-0.18	0.12	0.30	1/6
Indarch	EH4	-0.26	0.12	0.26	1/5	-0.18	0.12	0.27	1/5
MAC 88136	EL3	-0.21	0.08	0.25	1/9	-0.09	0.14	0.42	1/9
Tennasilm	L4	-0.39	-	0.23	1/2	-0.45	-	0.29	1/2
NWA 11880	R3.5-4	-0.33	0.09	0.27	2/8	-0.23	0.17	0.49	2/8
NWA 11754	Ureilite	-0.44	0.10	0.26	1/7	-0.29	0.28	0.73	1/7
NWA 11757	Ureilite	-0.32	0.07	0.17	1/6	-0.17	0.22	0.54	1/6
NWA 11890	Ureilite	-0.47	0.10	0.27	1/8	-0.35	0.07	0.21	1/8
NWA 11395	Howardite	-0.70	0.31	0.54	1/3	-0.62	0.06	0.10	1/3
NWA 12265	Eucrite	-0.38	0.28	0.62	1/5	-0.04	0.20	0.45	1/5
NWA 14443	Eucrite	-0.40	0.11	0.30	1/7	-0.22	0.10	0.25	1/7
NWA 8287	Acapulcoite	-0.22	0.08	0.28	1/10	-0.22	0.13	0.40	1/10
Djoua	Aubrite	-0.47	0.35	0.71	1/4	-0.10	0.35	0.70	1/4
Nantan	IAB complex	-0.20	0.15	0.27	1/3	-0.18	0.19	0.33	1/3
Toluca	IAB complex	-0.23	0.14	0.24	1/3	-0.23	0.10	0.18	1/3
BCR-2	Earth	-0.01	0.04	0.24	6/46	-0.01	0.06	0.42	6/46

Continued on next page.

Table S2. Continued.

Sample		Data normalized to $^{64}Zn/^{68}Zn$							
**Sample**		**$\varepsilon^{66/68}Zn$**	**2se**	**2sd**	**m/n**	**$\varepsilon^{67/68}Zn$**	**2se**	**2sd**	**m/n**
Orgueil	CI1	0.19	0.09	0.29	1/10	-0.14	0.07	0.23	1/10
Murchison	CM2	0.32	0.09	0.20	1/5	-0.15	0.09	0.29	1/5
Winchcombe	CM2	0.19	0.11	0.29	1/8	-0.16	0.10	0.28	1/8
NWA 13299	EH3	-0.16	0.08	0.26	2/11	0.14	0.17	0.55	2/11
MIL 07028	EH3	-0.27	0.08	0.20	1/6	-0.12	0.15	0.36	1/6
PCA 91238	EH3	-0.17	0.12	0.28	1/6	0.24	0.20	0.49	1/6
LAR 06252	EH3	-0.24	0.09	0.21	1/6	0.13	0.09	0.23	1/6
Indarch	EH4	-0.17	0.10	0.23	1/5	0.14	0.09	0.21	1/5
MAC 88136	EL3	-0.16	0.07	0.22	1/9	0.07	0.11	0.32	1/9
Tennasilm	L4	-0.11	-	0.06	1/2	0.34	-	0.22	1/2
NWA 11880	R3.5-4	-0.23	0.07	0.19	2/8	0.17	0.13	0.37	2/8
NWA 11754	Ureilite	-0.30	0.08	0.22	1/7	0.22	0.21	0.55	1/7
NWA 11757	Ureilite	-0.28	0.13	0.32	1/6	0.13	0.41	0.17	1/6
NWA 11890	Ureilite	-0.32	0.05	0.15	1/8	0.26	0.05	0.16	1/8
NWA 11395	Howardite	-0.25	0.26	0.46	1/3	0.53	0.06	0.11	1/3
NWA 12265	Eucrite	-0.35	0.32	0.72	1/5	0.04	0.15	0.34	1/5
NWA 14443	Eucrite	-0.26	0.11	0.29	1/7	0.18	0.07	0.20	1/7
NWA 8287	Acapulcoite	-0.25	0.07	0.21	1/10	0.16	0.10	0.30	1/10
Djoua	Aubrite	-0.39	0.27	0.54	1/4	0.08	0.27	0.55	1/4
Nantan	IAB complex	-0.15	0.19	0.32	1/3	0.13	0.15	0.15	1/3
Toluca	IAB complex	-0.10	0.09	0.16	1/3	0.17	0.08	0.14	1/3
BCR-2	Earth	0.00	0.04	0.28	6/46	0.00	0.05	0.32	6/46

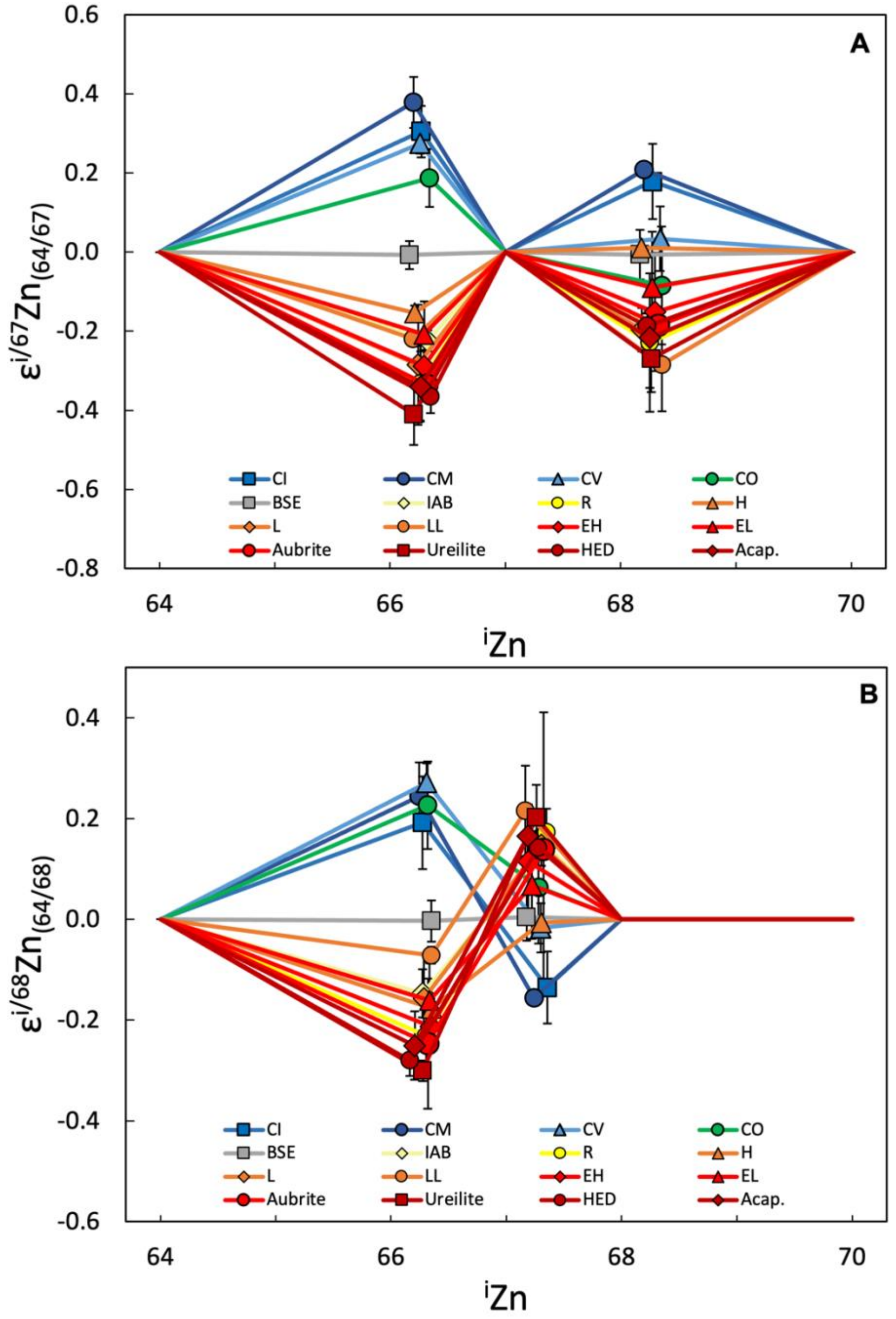

Fig. S1. Mean Zn isotope compositions for the meteorite groups and the BSE, in ε^{i}Zn notation. Same as Fig. 1 but data obtained using (A) ^{64}Zn/^{67}Zn and (B) ^{64}Zn/^{68}Zn for internal normalization.

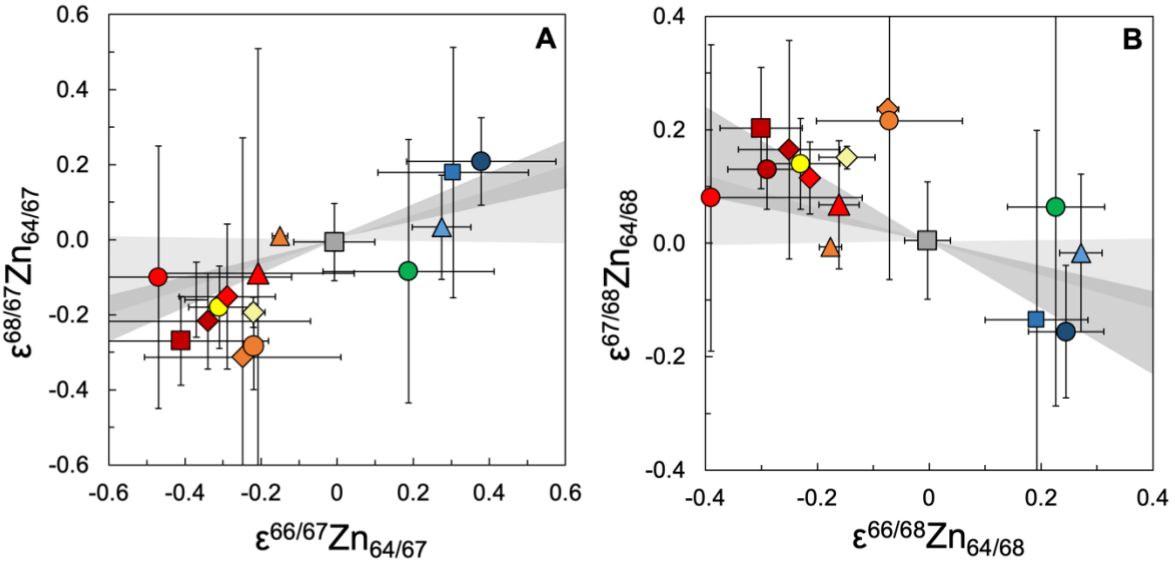

Fig. S2. Measured Zn isotope compositions in comparison to predicted nucleosynthetic isotope anomalies. Same as Fig. 2 but using the (A) $^{64}Zn/^{67}Zn$ and the (B) $^{64}Zn/^{68}Zn$ normalization ratios.

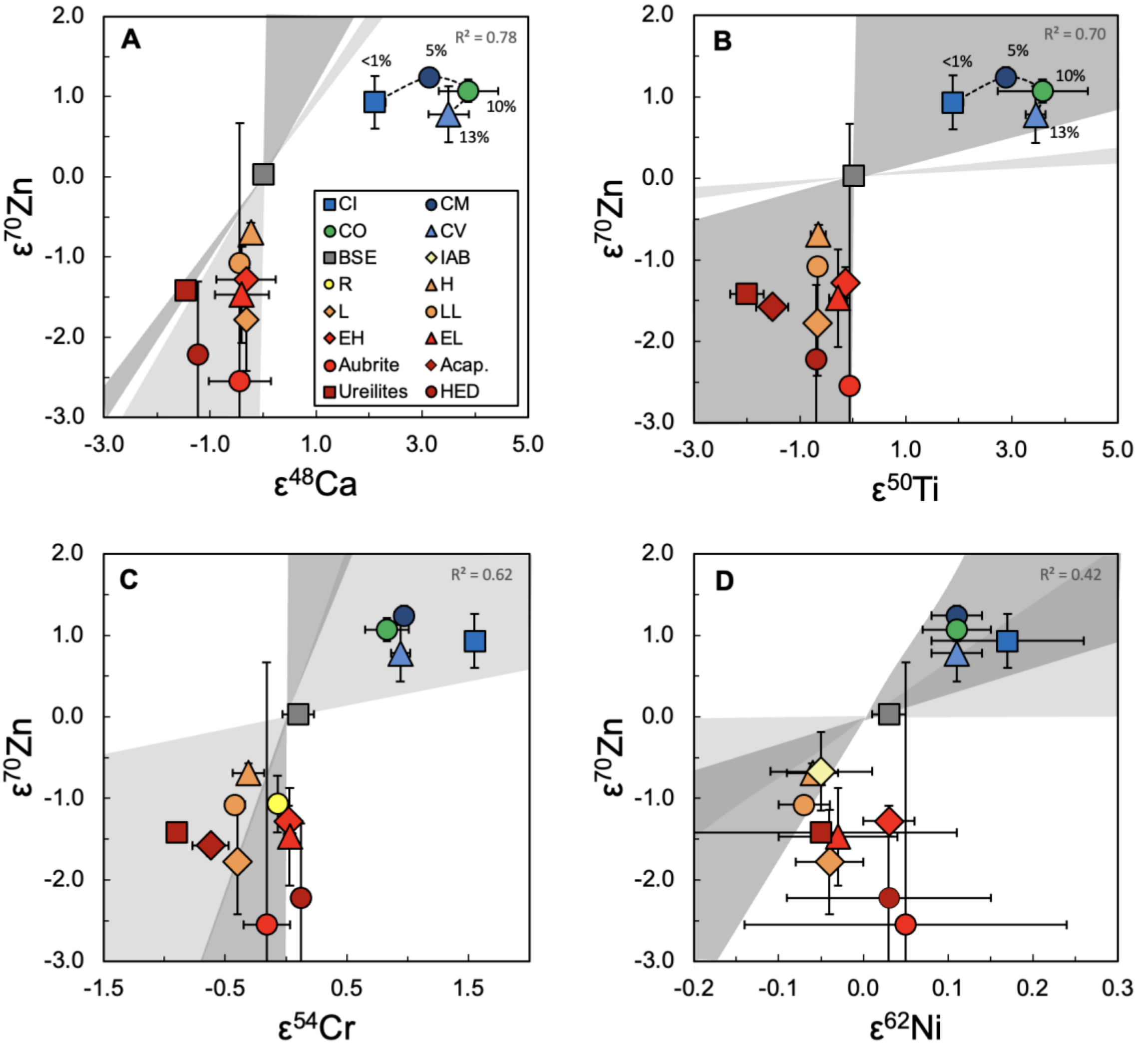

Fig. S3. Measured ^{70}Zn isotope anomalies versus isotope anomalies of other Fe-peak elements. Same as Fig. 3 but using ε^{70}Zn values instead of ε^{64}Zn.

Table S3. Summary of compiled isotope data used in the modelling.

Group		$\Delta^{17}O$	±	$\varepsilon^{48}Ca$	±	$\varepsilon^{50}Ti$	±	$\varepsilon^{54}Cr$	±	$\varepsilon^{64}Zn$	±
CI	CC	0.46	0.12	2.09	0.03	1.90	0.08	1.47	0.09	-0.93	0.20
CM	CC	-3.09	0.54	2.62	1.04	2.99	0.18	1.00	0.11	-1.15	0.20
CO	CC	-4.38	0.23	3.87	0.56	3.49	0.35	0.83	0.16	-0.57	0.22
CV	CC	-3.63	0.40	3.24	1.36	3.28	0.14	0.88	0.08	-0.83	0.08
H	NC	0.93	0.04	-0.04	0.36	-0.57	0.09	-0.37	0.03	0.47	0.06
L	NC	1.05	0.03	-0.26	0.08	-0.64	0.02	-0.34	0.10	0.81	0.34
LL	NC	1.20	0.05	-0.32	0.09	-0.67	0.03	-0.40	0.06	0.67	0.02
OC Mean		*1.06*	*0.22*	*-0.21*	*0.24*	*-0.62*	*0.08*	*-0.37*	*0.05*	*0.65*	*0.28*
EH	NC	-0.04	0.10	-0.20	0.23	-0.15	0.03	0.05	0.06	0.84	0.13
EL	NC	0.03	0.05	-0.25	0.13	-0.25	0.06	0.03	0.05	0.63	0.25
EC Mean		*-0.01*	*0.08*	*-0.22*	*0.05*	*-0.20*	*0.10*	*0.04*	*0.02*	*0.74*	*0.21*
Angrites	NC	-0.08	0.05	-1.06	0.18	-1.16	0.02	-0.41	0.06	0.89†	0.42
HEDs	NC	-0.29	0.07	-1.37	0.26	-1.24	0.03	-0.69	0.06	1.39	0.39
BSE		0.002	0.004	0.01	0.01	0.01	0.01	0.09	0.12	0.02	0.11
UM1	NC	1.06	0.22	-0.21	0.24	-0.62	0.08	-0.37	0.05	0.65	0.28
UM2	NC	-0.01	0.08	-0.22	0.05	-0.20	0.10	0.04	0.02	0.74	0.21

Group		$\varepsilon^{84}Sr$	±	$\varepsilon^{96}Zr$	±	$\varepsilon\ ^{30}Si$	±	$\delta^{30}Si$	±	$\delta^{25}Mg$	±
CI	CC	0.33	0.09	0.52	0.35	0.334	0.023	-0.44	0.17	-0.100	0.010
CM	CC	0.40	0.21	0.90	0.29	0.163	0.029	-0.50	0.05	-0.145	0.010
CO	CC	0.47	0.16	0.80	0.25	0.190	0.030	-0.46	0.07	-0.158	0.010
CV	CC	0.79	0.07	1.01	0.17	0.147	0.017	-0.47	0.07	-0.134‡	0.035
H	NC	0.03	0.40	0.48	0.19	-	-	-0.45	0.02	-0.145	0.025
L	NC	0.03	0.11	0.37	0.15	0.074	0.043	-0.45	0.04	-0.147§	0.005
LL	NC	-0.15	0.15	0.31	0.03	0.074	0.029	-0.43	0.04	-0.150	0.004
OC Mean		*-0.03*	*0.17*	*0.36*	*0.11*	*0.074*	*0.009*	*-0.45*	*0.02*	*-0.147*	*0.005*
EH	NC	-0.09	0.06	0.09	0.12	0.118	0.027	-0.72	0.06	-0.133	0.007
EL	NC	0.04	0.05	0.15	0.06	0.118	0.031	-0.58	0.03	-0.133	0.007
EC Mean		*-0.03*	*0.12*	*0.12*	*0.06*	*0.118*	*0.024*	*-0.65*	*0.13*	*-0.13*	*0.00*
Angrites	NC	0.00	0.09	0.50	0.05	-0.101	0.020	-0.33	0.06	-0.079	0.000
HEDs	NC	-0.07	0.09	0.41	0.09	-0.092	0.030	-0.43	0.03	-0.111	0.013
BSE		-0.07	0.15	0.05	0.05	0.013	0.025	-0.29	0.08	-0.121	0.005
UM1	NC	-0.03	0.17	0.36	0.11	0.074	0.00	-0.38	0.10	-0.095	0.032
UM2	NC	-0.03	0.12	0.12	0.06	0.118	0.001	-0.38	0.10	-0.095	0.032

All compiled data from which the group means were derived are available in the Data S1 file. †: Estimated by bivariant regression, see main text for details. For groups for which some of the data were not available, either CC (‡), or OC (§) means were used.

Table S4. Compiled concentration data for the elements and meteorite classes considered in the modelling.

Class	Bulk Earth	CI	CM	CO	CV	H
References	[18, 41, 42, 74-75]	[22, 28]	[22, 28]	[22, 28]	[22, 28]	[22, 23]
Na (%)	0.125 – 0.267	0.25 – 0.58	0.087 – 0.46	0.365 – 0.41	0.238 – 0.316	0.591 – 0.659
Mg (%)	13.9 – 15.4	9.5 – 10.5	11.5 – 12.4	10.8 – 13	12.5 – 14.5	13.8 – 14.4
Al (%)	1.41 – 1.63	0.80 – 0.86	1.1 – 1.24	1.11 – 1.43	1.42. -1.75	1.1 – 1.2
Si (%)	14.2 – 16.8	10.5	12.9	15.9	15.6	16.9
P ($\mu g\ g^{-1}$)	555 – 1920	845 – 1108	900 – 1108	1010 – 1060	990 – 1041	1080
Ca (%)	1.54 – 1.77	0.78 – 1.01	1.01 – 1.55	1.28 – 1.58	1.59 – 1.90	1.17 – 1.33
K ($\mu g\ g^{-1}$)	135 – 197	407 – 608	206 – 518	272 – 345	251 – 310	727 – 860
Ti ($\mu g\ g^{-1}$)	762 – 822	381 – 458	520 – 664	601 -656	753 – 980	600
Fe (%)	29.6 – 32.7	18.2 – 21.3	21 – 23.6	21.7 – 24.8	22.2 – 23.8	26.4 – 29.7
Mn ($\mu g\ g^{-1}$)	750 – 2594	1900 – 2061	1700 – 1920	1475 – 1650	1334 – 1465	2180 – 2430
O (%)	20.12 – 31.67	46	43.2	37	37	35.7
Cr ($\mu g\ g^{-1}$)	3300 – 4466	2575 – 3001	3033 – 3478	3111 – 3550	3253 – 3603	3460 – 3870
Zn ($\mu g\ g^{-1}$)	40 – 74	312 – 391	182 – 214	100 – 119	112 – 127	41 – 55
Zr ($\mu g\ g^{-1}$)	6.32 – 7.1	3.87 – 5	4.48 – 10.1	4.92 – 12.7	5.85 – 15.3	10
Sr ($\mu g\ g^{-1}$)	13 – 14.7	3.8-4	8	7.8	8.3	6.3

Continued on next page.

Table S4. Continued.

Class	L	LL	EH	EL	EPB	APB	UM1/2
References	[22, 23]	[22, 23]	[22, 24]	[22, 24]	[26, 76-80]	[27, 72, 76, 80]	
Na (%)	0.58 - 0.731	0.576 - 0.728	0.474 - 0.78	0.51 - 0.63	0.104 - 0.54	0.02	0.02 - 0.54
Mg (%)	14.1 - 15.2	14.7 - 15.5	9.6 11.9	13.3 - 14.6	15 - 18.4	12.0	12.0 - 18.4
Al (%)	1.17 - 1.26	1.16 - 1.31	0.74 - 0.87	1 - 1.14	1.2 - 1.9	1.43	1.2 - 1.43
Si (%)	18.5	18.9	16.7	18.6	20.9 - 21.5	13.1	13.1 - 21.5
P (µg g^{-1})	950	850	2000	1170	1877	938	938 - 1877
Ca (%)	1.23 - 1.38	1.18 - 1.39	0.65 - 1	0.85 - 1.2	1.3 - 2.14	1.50	1.3 - 2.14
K (µg g^{-1})	505 - 950	637 - 922	402 - 1240	600 - 845	30 - 54	12	12 - 54
Ti (µg g^{-1})	630	620	450	580	347.4 - 830	599.0	347.4 - 830
Fe (%)	20.3 - 24.5	17.6 - 21.2	24.9 - 31.5	19.5 - 27	11.1 - 29.2	19.4	11.1 - 29.2
Mn (µg g^{-1})	2410 - 2700	2490 - 2720	1820 - 2650	1120 - 2560	1394 - 4336	1549	1394 - 4336
O (%)	37.7	40	28	31	34.48 - 42.65	34.5	34.48 - 42.65
Cr (µg g^{-1})	3630 - 3940	3630 - 4060	2820 - 3870	2460 - 3870	1916 - 4611	2737	1916 - 4611
Zn (µg g^{-1})	43.5 - 63	41 - 60.6	21 - 342	13 - 26	0.11 – 8.9[a]	5 – 12*	0.11 - 12
Zr (µg g^{-1})	11.1	11.1	7.2	8.2	7.9	6.32 – 7.1†	6.32 – 7.9
Sr (µg g^{-1})	5.9	5.9	4.9	5.2	16.6	10	10 – 16.6

Compiled from (*18, 22-24, 26-28, 72, 74-80*). *: no estimates for bulk parent body were available, so both the APB and EPB were allowed to vary within measured values for relevant meteorites; †: no estimates for bulk parent body available, so allowed to vary within estimated BE range.

Table S5. Collector configurations that were used for the Zn isotope measurements, abundances of the isotopes of Zn as well as spectral interferences from isobars, Ba^{2+} and the most relevant molecular ions.

Faraday collector	L4	L2	Ax	H1	H2	H4	H6	H7	H8	H10
**Collector configuration M64**	^{62}Ni	^{64}Zn			^{66}Zn	^{67}Zn	^{68}Zn	$^{137}Ba^{2+}$	^{70}Zn	
Isotope abundance Zn		49.2%			27.7%	4.0%	18.5%		0.6%	
Isotope abundance Ni	3.6%	0.1%								
Isotope abundance Ba^{2+} ions					0.1%	2.4%	7.9%	11.2%		
Isotope abundance Ge									20.5%	
**Collector configuration M70**	^{66}Zn	^{67}Zn	^{68}Zn	$^{137}Ba^{2+}$		^{70}Zn			^{72}Ge	^{73}Ge
Isotope abundance Zn	27.7%	4.0%	18.5%			0.6%				
Isotope abundance Ge						20.5%			27.5%	7.8%
Isotope abundance Ba^{2+} ions	0.1%	2.4%	7.9%	11.2%						

Table S6. ε^{64}Zn values for angrites estimated by bivariate regressions. Three regressions were performed based on correlations between ε^{64}Zn and ε^{48}Ca, ε^{50}Ti, and ε^{54}Cr. The measured ε^{48}Ca, ε^{50}Ti, and ε^{54}Cr values of angrites, and the slope (m) and intercept (b) of the regressions were used to estimate ε^{64}Zn values for angrites. The three results were averaged, and all uncertainties were propagated, resulting in ε^{64}Zn = 0.89 ± 0.42 for angrites.

	^{48}Ca	^{50}Ti	^{54}Cr
$\varepsilon^{i}E_{Angrites}$	-1.06 ± 0.33	-1.18 ± 0.08	-0.43 ± 0.06
m	-0.402 ± 0.063	-0.421 ± 0.065	-1.210 ± 0.172
b	0.397 ± 0.081	0.387 ± 0.078	0.437 ± 0.085
$\varepsilon^{64}Zn_{Angrites}$	0.82 ± 0.35	0.88 ± 0.13	0.96 ± 0.20

Data S1. Compiled isotope data. Isotope data for the BSE and meteorite groups included in the modelling. Adapted from Dauphas et al. (*45*).

REFERENCES AND NOTES

1. N. Dauphas, J. H. Chen, J. Zhang, D. A. Papanastassiou, A. M. Davis, C. Travaglio, Calcium-48 isotopic anomalies in bulk chondrites and achondrites: Evidence for a uniform isotopic reservoir in the inner protoplanetary disk. *Earth Planet. Sci. Lett.* **407**, 96–108 (2014).

2. A. Trinquier, T. Elliott, D. Ulfbeck, C. Coath, A. N. Krot, M. Bizzarro, Origin of nucleosynthetic isotope heterogeneity in the solar protoplanetary disk. *Science* **324**, 374–376 (2009).

3. A. Trinquier, J. L. Birck, C. J. Allegre, Widespread Cr-54 heterogeneity in the inner solar system. *Astrophys. J.* **655**, 1179–1185 (2007).

4. J. Render, G. A. Brennecka, S.-J. Wang, L. E. Wasylenki, T. Kleine, A distinct nucleosynthetic heritage for early solar system solids recorded by Ni isotope signatures. *Astrophys. J.* **862**, 26 (2018).

5. P. S. Savage, F. Moynier, M. Boyet, Zinc isotope anomalies in primitive meteorites identify the outer solar system as an important source of Earth's volatile inventory. *Icarus* **386**, 115172 (2022).

6. T. Steller, C. Burkhardt, C. Yang, T. Kleine, Nucleosynthetic zinc isotope anomalies reveal a dual origin of terrestrial volatiles. *Icarus* **386**, 115171 (2022).

7. R. Martins, S. Kuthning, B. J. Coles, K. Kreissig, M. Rehkämper, Nucleosynthetic isotope anomalies of zinc in meteorites constrain the origin of Earth's volatiles. *Science* **379**, 369–372 (2023).

8. W. Akram, M. Schönbächler, S. Bisterzo, R. Gallino, Zirconium isotope evidence for the heterogeneous distribution of s-process materials in the solar system. *Geochim. Cosmochim. Acta* **165**, 484–500 (2015).

9. F. Moynier, J. M. D. Day, W. Okui, T. Yokoyama, A. Bouvier, R. J. Walker, F. A. Podosek, Planetary-scale strontium isotopic heterogeneity and the age of volatile depletion of early Solar System materials. *Astrophys. J.* **758**, 45 (2012).

10. N. Dauphas, B. Marty, L. Reisberg, Molybdenum evidence for inherited planetary scale isotope heterogeneity of the protosolar nebula. *Astrophys. J.* **565**, 640–644 (2002).

11. M. Fischer-Gödde, B.-M. Elfers, C. Münker, K. Szilas, W. D. Maier, N. Messling, T. Morishita, M. Van Kranendonk, H. Smithies, Ruthenium isotope vestige of Earth's pre-late-veneer mantle preserved in Archaean rocks. *Nature* **579**, 240–244 (2020).

12. K. Lodders, An oxygen isotope mixing model for the accretion and composition of rocky planets. *Space Sci. Rev.* **92**, 341–354 (2000).

13. N. Dauphas, The isotopic nature of the Earth's accreting material through time. *Nature* **541**, 521–524 (2017).

14. C. Fitoussi, B. Bourdon, Silicon isotope evidence against an enstatite chondrite Earth. *Science* **335**, 1477–1480 (2012).

15. R. C. Hin, C. D. Coath, P. J. Carter, F. Nimmo, Y.-J. Lai, P. A. E. Pogge von Strandmann, M. Willbold, Z. M. Leinhardt, M. J. Walter, T. Elliott, Magnesium isotope evidence that accretional vapour loss shapes planetary compositions. *Nature* **549**, 511–515 (2017).

16. E. A. Pringle, F. Moynier, P. S. Savage, J. Badro, J.-A. Barrat, Silicon isotopes in angrites and volatile loss in planetesimals. *Proc. Natl. Acad. Sci. U.S.A.* **111**, 17029–17032 (2014).

17. K. Lodders, Solar system abundances and condensation temperatures of the elements. *Astrophys. J.* **591**, 1220–1247 (2003).

18. W. F. McDonough, in *Treatise on Geochemistry (Second Edition)*, H. D. Holland, K. K. Turekian, Eds. (Elsevier, 2014), pp. 559–577.

19. G. J. Taylor, The bulk composition of Mars. *Geochemistry* **73**, 401–420 (2013).

20. P. N. Peplowski, L. G. Evans, S. A. Hauck II, T. J. McCoy, W. V. Boynton, J. L. Gillis-Davis, D. S. Ebel, J. O. Goldsten, D. H. Hamara, D. J. Lawrence, R. L. McNutt Jr., L. R. Nittler, S. C. Solomon, E. A. Rhodes, A. L. Sprague, R. D. Starr, K. R. Stockstill-Cahill, Radioactive elements

on Mercury's surface from MESSENGER: Implications for the planet's formation and evolution. *Science* **333**, 1850–1852 (2011).

21. H. S. C. O'Neill, The origin of the Moon and the early history of the Earth—A chemical model. Part 2: The Earth. *Geochim. Cosmochim. Acta* **55**, 1159–1172 (1991).

22. J. T. Wasson, G. W. Kallemeyn, Compositions of chondrites. *Phil. Trans. R. Soc. Lond. Ser. A* **325**, 535–544 (1988).

23. G. W. Kallemeyn, A. E. Rubin, D. Wang, J. T. Wasson, Ordinary chondrites: Bulk compositions, classification, lithophile-element fractionations and composition-petrographic type relationships. *Geochim. Cosmochim. Acta* **53**, 2747–2767 (1989).

24. G. W. Kallemeyn, J. T. Wasson, Composition of enstatite (EH3, EH4,5 and EL6) chondrites: Implications regarding their formation. *Geochim. Cosmochim. Acta* **50**, 2153–2164 (1986).

25. J. T. Wasson, C. L. Chou, R. W. Bild, P. A. Baedecker, Classification of and elemental fractionation among ureilites. *Geochim. Cosmochim. Acta* **40**, 1449–1458 (1976).

26. R. C. Paniello, F. Moynier, P. Beck, J.-A. Barrat, F. A. Podosek, S. Pichat, Zinc isotopes in HEDs: Clues to the formation of 4-Vesta, and the unique composition of Pecora Escarpment 82502. *Geochim. Cosmochim. Acta* **86**, 76–87 (2012).

27. P. H. Warren, G. W. Kallemeyn, Geochemistry of the LEW87051 angrite and other basaltic achondrites. *Lunar Planet. Sci.* **XXI**, 1295 (1990).

28. N. Braukmüller, F. Wombacher, D. C. Hezel, R. Escoube, C. Münker, The chemical composition of carbonaceous chondrites: Implications for volatile element depletion, complementarity and alteration. *Geochim. Cosmochim. Acta* **239**, 17–48 (2018).

29. B. Marty, The origins and concentrations of water, carbon, nitrogen and noble gases on Earth. *Earth Planet. Sci. Lett.* **313-314**, 56–66 (2012).

30. F. Albarède, C. Ballhaus, J. Blichert-Toft, C.-T. Lee, B. Marty, F. Moynier, Q.-Z. Yin, Asteroidal impacts and the origin of terrestrial and lunar volatiles. *Icarus* **222**, 44–52 (2013).

31. N. X. Nie, D. Wang, Z. A. Torrano, R. W. Carlson, C. M. O'D. Alexander, A. Shahar, Meteorites have inherited nucleosynthetic anomalies of potassium-40 produced in supernovae. *Science* **379**, 372–376 (2023).

32. C. Fitoussi, B. Bourdon, X. Y. Wang, The building blocks of Earth and Mars: A close genetic link. *Earth Planet. Sci. Lett.* **434**, 151–160 (2016).

33. M. Schiller, M. Bizzarro, V. A. Fernandes, Isotopic evolution of the protoplanetary disk and the building blocks of Earth and the Moon. *Nature* **555**, 507–510 (2018).

34. I. J. Onyett, M. Schiller, G. V. Makhatadze, Z. Deng, A. Johansen, M. Bizzarro, Silicon isotope constraints on terrestrial planet accretion. *Nature* **619**, 539–544 (2023).

35. S. Wanajo, H.-T. Janka, B. Müller, Electron-capure supernovae as origin of ^{48}Ca. *Astrophys. J. Lett.* **767**, L26 (2013).

36. S. E. Woosley, Neutron-rich nucleosynthesis in carbon deflagration supernovae. *Astrophys. J.* **476**, 801–810 (1997).

37. R. C. J. Steele, C. D. Coath, M. Regelous, S. Russell, T. Elliott, Neutron-poor nickel isotope anomalies in meteorites. *Astrophys. J.* **758**, 59 (2012).

38. N. Dauphas, L. Remusat, J. H. Chen, M. Roskosz, D. A. Papanastassiou, J. Stodolna, Y. Guan, C. Ma, J. M. Eiler, Neutron-rich chromium isotope anomalies in supernova nanoparticles. *Astrophys. J.* **720**, 1577–1591 (2010).

39. K. Lodders, B. Fegley, *The Planetary Scientist's Companion* (Oxford Univ. Press, 1998).

40. B. Mahan, J. Siebert, I. Blanchard, S. Borensztajn, J. Badro, F. Moynier, Constraining compositional proxies for Earth's accretion and core formation through high pressure and high temperature Zn and S metal-silicate partitioning. *Geochim. Cosmochim. Acta* **235**, 21–40 (2018).

41. H. S. Wang, C. H. Lineweaver, T. R. Ireland, The elemental abundances (with uncertainties) of the most Earth-like planet. *Icarus* **299**, 460–474 (2018).

42. W. F. McDonough, in *Meteorites, Comets, and Planets*, A. M. Davis, Ed. (Elsevier, 2005), pp. 547–568.

43. B. Mahan, J. Siebert, E. A. Pringle, F. Moynier, Elemental partitioning and isotopic fractionation of Zn between metal and silicate and geochemical estimation of the S content of the Earth's core. *Geochim. Cosmochim. Acta* **196**, 252–270 (2017).

44. L. J. Bridgestock, H. Williams, M. Rehkämper, F. Larner, M. D. Giscard, S. Hammond, B. Coles, R. Andreasen, B. J. Wood, K. J. Theis, C. L. Smith, G. K. Benedix, M. Schönbächler, Unlocking the zinc isotope systematics of iron meteorites. *Earth Planet. Sci. Lett.* **400**, 153–164 (2014).

45. N. Dauphas, T. Hopp, D. Nesvorný, Bayesian inference on the isotopic building blocks of Mars and Earth. *Icarus* **408**, 115805 (2024).

46. A. Morbidelli, J. Chambers, J. I. Lunine, J. M. Petit, F. Robert, G. B. Valsecchi, K. E. Cyr, Source regions and timescales for the delivery of water to the Earth. *Meteorit. Planet. Sci.* **35**, 1309–1320 (2000).

47. C. Burkhardt, F. Spitzer, A. Morbidelli, G. Budde, J. H. Render, T. S. Kruijer, T. Kleine, Terrestrial planet formation from lost inner solar system material. *Sci. Adv.* **7**, eabj7601 (2021).

48. D. Clayton, *Isotopes in the Cosmos: Hydrogen to Gallium* (Cambridge Univ. Press, 2003).

49. N. Braukmüller, F. Wombacher, C. Funk, C. Münker, Earth's volatile element depletion pattern inherited from a carbonaceous chondrite-like source. *Nat. Geosci.* **12**, 564–568 (2019).

50. P. H. Warren, Stable-isotopic anomalies and the accretionary assemblage of the Earth and Mars: A subordinate role for carbonaceous chondrites. *Earth Planet. Sci. Lett.* **311**, 93–100 (2011).

51. J. Render, M. Fischer-Gödde, C. Burkhardt, T. Kleine, The cosmic molybdenum-neodymium isotope correlation and the building material of the Earth. *Geochem. Perspect. Lett.* **3**, 170–178. (2017).

52. R. Wolf, M. Ebihara, G. R. Richter, E. Anders, Aubrites and diogenites: Trace element clues to their origin. *Geochim. Cosmochim. Acta* **47**, 2257–2270 (1983).

53. P. S. Savage, F. Moynier, Silicon isotopic variation in enstatite meteorites: Clues to their origin and Earth-forming material. *Earth Planet. Sci. Lett.* **361**, 487–496 (2013).

54. G. Budde, C. Burkhardt, T. Kleine, Molybdenum isotopic evidence for the late accretion of outer Solar System material to Earth. *Nat. Astron.* **3**, 736–741 (2019).

55. R. Martins, M. Chaussidon, Z. Deng, F. Pignatale, F. Moynier, A condensation origin for the mass-dependent silicon isotopic variations in Allende components: Implications for complementarity. *Earth Planet. Sci. Lett.* **554**, 116678 (2021).

56. R. B. Georg, A. N. Halliday, E. Schauble, B. C. Reynolds, Silicon in the Earth's core. *Nature* **447**, 1102–1106 (2007).

57. J. B. Creech, F. Moynier, Tin and zinc stable isotope characterisation of chondrites and implications for early Solar System evolution. *Chem. Geol.* **511**, 81–90 (2019).

58. M. A. Fehr, S. J. Hammond, I. J. Parkinson, Tellurium stable isotope fractionation in chondritic meteorites and some terrestrial samples. *Geochim. Cosmochim. Acta* **222**, 17–33 (2018).

59. J. L. Hellmann, T. Hopp, C. Burkhardt, T. Kleine, Origin of volatile element depletion among carbonaceous chondrites. *Earth Planet. Sci. Lett.* **549**, 116508 (2020).

60. J. L. Hellmann, T. Hopp, C. Burkhardt, H. Becker, M. Fischer-Gödde, T. Kleine, Tellurium isotope cosmochemistry: Implications for volatile fractionation in chondrite parent bodies and origin of the late veneer. *Geochim. Cosmochim. Acta* **309**, 313–328 (2021).

61. N. Dauphas, M. Chaussidon, A perspective from extinct radionuclides on a young stellar object: The Sun and its accretion disk. *Annu. Rev. Earth Planet. Sci.* **39**, 351–386 (2011).

62. M. E. Newcombe, S. G. Nielsen, L. D. Peterson, J. Wang, C. M. O'D. Alexander, A. R. Sarafian, K. Shimizu, L. R. Nittler, A. J. Irving, Degassing of early-formed planetesimals restricted water delivery to Earth. *Nature* **615**, 854–857 (2023).

63. B. Carry, Density of asteroids. *Planet. Space Sci.* **73**, 98–118 (2012).

64. T. Lichtenberg, G. J. Golabek, R. Burn, M. R. Meyer, Y. Alibert, T. V. Gerya, C. Mordasini, A water budget dichotomy of rocky protoplanets from ^{26}Al-heating. *Nat. Astron.* **3**, 307–313 (2019).

65. E. D. Young, Inheritance of solar short- and long-lived radionuclides from molecular clouds and the unexceptional nature of the solar system. *Earth Planet. Sci. Lett.* **392**, 16–27 (2014).

66. M. Reiter, Observational constraints on the likelihood of ^{26}Al in planet-forming environments. *Astron. Astrophys.* **644**, L1 (2020).

67. K. J. Walsh, A. Morbidelli, S. N. Raymond, D. P. O'Brien, A. M. Mandell, Populating the asteroid belt from two parent source regions due to the migration of giant planets—"The Grand Tack". *Meteorit. Planet. Sci.* **47**, 1941–1947 (2012).

68. M. Schönbächler, R. W. Carlson, M. F. Horan, T. D. Mock, E. H. Hauri, Heterogeneous accretion and the moderately volatile element budget of Earth. *Science* **328**, 884–887 (2010).

69. M. I. Varas-Reus, S. König, A. Yierpan, J.-P. Lorand, R. Schoenberg, Selenium isotopes as tracers of a late volatile contribution to Earth from the outer Solar System. *Nat. Geosci.* **12**, 779–782 (2019).

70. Z. Wang, H. Becker, Ratios of S, Se and Te in the silicate Earth require a volatile-rich late veneer. *Nature*, **499** (7458), 328–331 (2013).

71. P. Frossard, C. Israel, A. Bouvier, M. Boyet, Earth's composition was modified by collisional erosion. *Science* **377**, 1529–1532 (2022).

72. P. H. Warren, G. W. Kallemeyn, Angrites: A volatile-rich variety of asteroidal basalt (except for alkalis and gallium!). *Meteoritics* **30**, 593 (1995).

73. C. Wu, J. Z. Yu, Evaluation of linear regression techniques for atmospheric applications: The importance of appropriate weighting. *Atmos. Meas. Tech.* **11**, 1233–1250 (2018).

74. J. S. Kargel, J. S. Lewis, The composition and early evolution of Earth. *Icarus* **105**, 1–25 (1993).

75. J. W. Morgan, E. Anders, Chemical composition of Earth, Venus, and Mercury. *Proc. Natl. Acad. Sci. U.S.A.* **77**, 6973–6977 (1980).

76. C. Liebske, A. Khan, On the principal building blocks of Mars and Earth. *Icarus* **322**, 121–134 (2019).

77. G. Dreibus, H. Kruse, B. Spettel, H. Waenke, The bulk composition of the moon and the eucrite parent body. *Proc. Lunar Sci. Conf.* **8**, 211–227 (1977).

78. H. O. Ashcroft, B. J. Wood, An experimental study of partial melting and fractional crystallization on the HED parent body. *Meteorit. Planet. Sci.* **50**, 1912–1924 (2015).

79. J. W. Morgan, H. Higuchi, H. Takahashi, J. Hertogen, A "chondritic" eucrite parent body: Inference from trace elements. *Geochim. Cosmochim. Acta* **42**, 27–38 (1978).

80. A. J. Jurewicz, D. W. Mittlefehldt, J. H. Jones, Partial melting of the allende (CV3) meteorite: Implications for origins of basaltic meteorites. *Science* **252**, 695–698 (1991).
